# Analysis of MRI and CT-based radiomics features for personalized treatment in locally advanced rectal cancer and external validation of published radiomics models

**DOI:** 10.1038/s41598-022-13967-8

**Published:** 2022-06-17

**Authors:** Iram Shahzadi, Alex Zwanenburg, Annika Lattermann, Annett Linge, Christian Baldus, Jan C. Peeken, Stephanie E. Combs, Markus Diefenhardt, Claus Rödel, Simon Kirste, Anca-Ligia Grosu, Michael Baumann, Mechthild Krause, Esther G. C. Troost, Steffen Löck

**Affiliations:** 1 OncoRay-National Center for Radiation Research in Oncology, Faculty of Medicine and University Hospital Carl Gustav Carus, Technische Universität Dresden, Helmholtz-Zentrum Dresden-Rossendorf, Dresden, Germany; 2 German Cancer Consortium (DKTK) partner site Dresden, Germany and German Cancer Research Center (DKFZ), Heidelberg, Germany; 3 German Cancer Research Center (DKFZ), Heidelberg, Germany; 4 National Center for Tumor Diseases (NCT), Partner Site Dresden, Dresden, Germany; 5 Department of Radiotherapy and Radiation Oncology, Faculty of Medicine and University Hospital Carl Gustav Carus, Technische Universität Dresden, Dresden, Germany; 6 Department of Radiology, Faculty of Medicine and University Hospital Carl Gustav Carus, Technische Universität Dresden, Dresden, Germany; 7 German Cancer Consortium (DKTK) partner site Munich, Germany and German Cancer Research Center (DKFZ), Heidelberg, Germany; 8 Department of Radiation Oncology, Klinikum rechts der Isar, Technische Universität München, München, Germany; 9 Institute of Radiation Medicine (IRM), Department of Radiation Sciences (DRS), Helmholtz Zentrum München, Neuherberg, Germany; 10 Department of Radiotherapy and Oncology, Goethe-University Frankfurt, Frankfurt am Main, Germany; 11 German Cancer Consortium (DKTK) partner site Frankfurt, Germany and German Cancer Research Center (DKFZ), Heidelberg, Germany; 12 Frankfurt Cancer Institute, Frankfurt, Germany; 13 Department of Radiation Oncology, Medical Center, Faculty of Medicine, University of Freiburg, Freiburg, Germany; 14 German Cancer Consortium (DKTK) partner site Freiburg, Germany and German Cancer Research Center (DKFZ), Heidelberg, Germany; 15 Helmholtz-Zentrum Dresden-Rossendorf, Institute of Radiooncology-OncoRay, Dresden, Germany

**Keywords:** Prognostic markers, Rectal cancer, Cancer imaging, Radiotherapy

## Abstract

Radiomics analyses commonly apply imaging features of different complexity for the prediction of the endpoint of interest. However, the prognostic value of each feature class is generally unclear. Furthermore, many radiomics models lack independent external validation that is decisive for their clinical application. Therefore, in this manuscript we present two complementary studies. In our modelling study, we developed and validated different radiomics signatures for outcome prediction after neoadjuvant chemoradiotherapy (nCRT) in patients with locally advanced rectal cancer (LARC) based on computed tomography (CT) and T2-weighted (T2w) magnetic resonance (MR) imaging datasets of 4 independent institutions (training: 122, validation 68 patients). We compared different feature classes extracted from the gross tumour volume for the prognosis of tumour response and freedom from distant metastases (FFDM): morphological and first order (MFO) features, second order texture (SOT) features, and Laplacian of Gaussian (LoG) transformed intensity features. Analyses were performed for CT and MRI separately and combined. Model performance was assessed by the area under the curve (AUC) and the concordance index (CI) for tumour response and FFDM, respectively. Overall, intensity features of LoG transformed CT and MR imaging combined with clinical T stage (cT) showed the best performance for tumour response prediction, while SOT features showed good performance for FFDM in independent validation (AUC = 0.70, CI = 0.69). In our external validation study, we aimed to validate previously published radiomics signatures on our multicentre cohort. We identified relevant publications on comparable patient datasets through a literature search and applied the reported radiomics models to our dataset. Only one of the identified studies could be validated, indicating an overall lack of reproducibility and the need of further standardization of radiomics before clinical application.

## Introduction

Personalized treatment strategies can play an essential role in oncological patient management as they are expected to improve outcomes of patient populations with heterogeneous treatment response. In particular, for patients with locally advanced rectal cancer (LARC), the response to neoadjuvant chemoradiotherapy (nCRT) varies widely, ranging from pathological complete response (pCR) with no viable remaining tumour cells to persisting disease (pathological non-responders: pNRs)^1^. There is increased interest in the application of organ-preserving and low-morbidity surgeries or watch-and-wait strategies, for patients with clinical complete response (cCR) after neoadjuvant or total neoadjuvant CRT^2,3^. These strategies require validated biomarkers that allow for an early and accurate identification of this patient population. Several studies have been analysing molecular data, such as gene expressions, mutations, and single nucleotide polymorphisms as potential biomarkers of response to nCRT in LARC^4–6^. The inclusion of non-invasive biomarkers from clinical imaging may further increase the robustness and accuracy of corresponding prognostic models.

Radiomic analyses employ classical statistics and modern machine learning algorithms to identify biomarkers based on multimodality imaging and have shown a great potential for treatment outcome prediction in different cancer entities^7–9^. For predicting patient’s response to nCRT and long-term outcomes including freedom from distant metastases (FFDM) and overall survival in LARC, radiomics models were widely developed on features extracted from T2-weighted (T2w) magnetic resonance imaging (MRI)^10–15^, and multiparametric MRI (mpMRI)^16–20^. Few studies have considered radiomic features extracted from computed tomography (CT) imaging^21,22^, positron emission tomography (PET)^23,24^, or a combination of CT and MRI features^25^. Although the results of these analyses are encouraging, important aspects, such as assessing feature robustness, were not always considered and external validation was rarely performed.

One key challenge in radiomics is the selection of features that correlate well with the endpoint of interest^26^. Feature classes of different complexity are commonly extracted: (i) morphological features that describe the shape of the region of interest (ROI), (ii) first-order features (FO) that describe the voxel intensity distribution, (iii) second-order texture features (SOT) that describe statistical inter-relationships between neighbouring voxels, and (iv) higher order features, where (i)–(iii) are extracted after applying transformations on the base images. In several studies, morphological and first order (MFO) features extracted from pre-treatment T2w MRI^12,16,27,28^ had a high association to treatment response in LARC. Other studies considered SOT features only^13,29,30^ or in combination with MFO and SOT features^11,14,15,17^. However, it is generally unclear which feature classes are more relevant and generalizable for predicting treatment outcomes in patients with locally advanced rectal cancer.

In this manuscript, we present two studies related to the described open questions of radiomics for LARC: (i) In the modelling study, we identified and independently validated novel radiomic signatures for the prognosis of tumour response to nCRT and FFDM in patients with LARC using a multicentre retrospective cohort of the German Cancer Consortium—Radiation Oncology Group (DKTK-ROG). In particular, we investigated the prognostic value of different feature classes and developed multimodal radiomics signatures combining pre-treatment CT and T2w MRI with clinical characteristics. (ii) In the external validation study, we aimed to validate radiomics signatures that were previously developed by other researchers to predict tumour response to nCRT or FFDM in LARC using our multicentre data.

## Methods

### Patient data

In this multicentre retrospective study, data of 190 patients were collected from four partner sites within the DKTK-ROG and divided into training and validation data based on the site (122 and 68 patients, respectively). Ninety-four out of 122 patients of the training data were treated at the University Hospital Carl Gustav Carus Dresden between 2006 and 2014. The remaining 28 patients were treated at the Klinikum rechts der Isar Munich between 2007 and 2013. In the validation data, 12 out of 68 patients were treated at the University Hospital Freiburg between 2008 and 2013, while the remaining 56 patients were treated at the University Hospital Frankfurt between 2007 and 2015. All patients had a histopathologically confirmed diagnosis of LARC and underwent nCRT followed by surgery. Additional inclusion criteria for our study were the availability of pre-treatment T2w MRI, treatment planning CT with sufficient image quality (e.g. without strong streaking artifacts, patient motion or scanner distortions), and endpoint information. Ethical approval for the multicentre retrospective analyses was obtained from the Ethics Committee at the Technische Universität Dresden, Germany (BO-EK-385082020). The requirement for individual informed consent was waived owing to the retrospective nature of the study.

The considered endpoints were tumour response to nCRT and freedom from distant metastases (FFDM). Tumour response was determined by expert pathologists from the work-up of the surgical specimens. The patients were stratified into two groups based on the tumour regression grade (TRG): responders (corresponding to TRG 3 and 4, labelled as 1) and non-responders (corresponding to TRG 0–2, labelled as 0) following Dworak et al.^31^. For the external validation study, where we aimed to validate radiomic signatures from the literature, patients were stratified to match the stratification indicated in the respective manuscript. A detailed description of the TRG is presented in Supplementary Table S1. The survival endpoint FFDM was calculated from the first day of nCRT to the day of event or censoring. For patients with observed distant metastases, the event time was accompanied by an event indicator variable of 1, whereas for patients without an event, the last follow-up time was used together with an event indicator variable of 0.

### Study design

**Figure 1:**
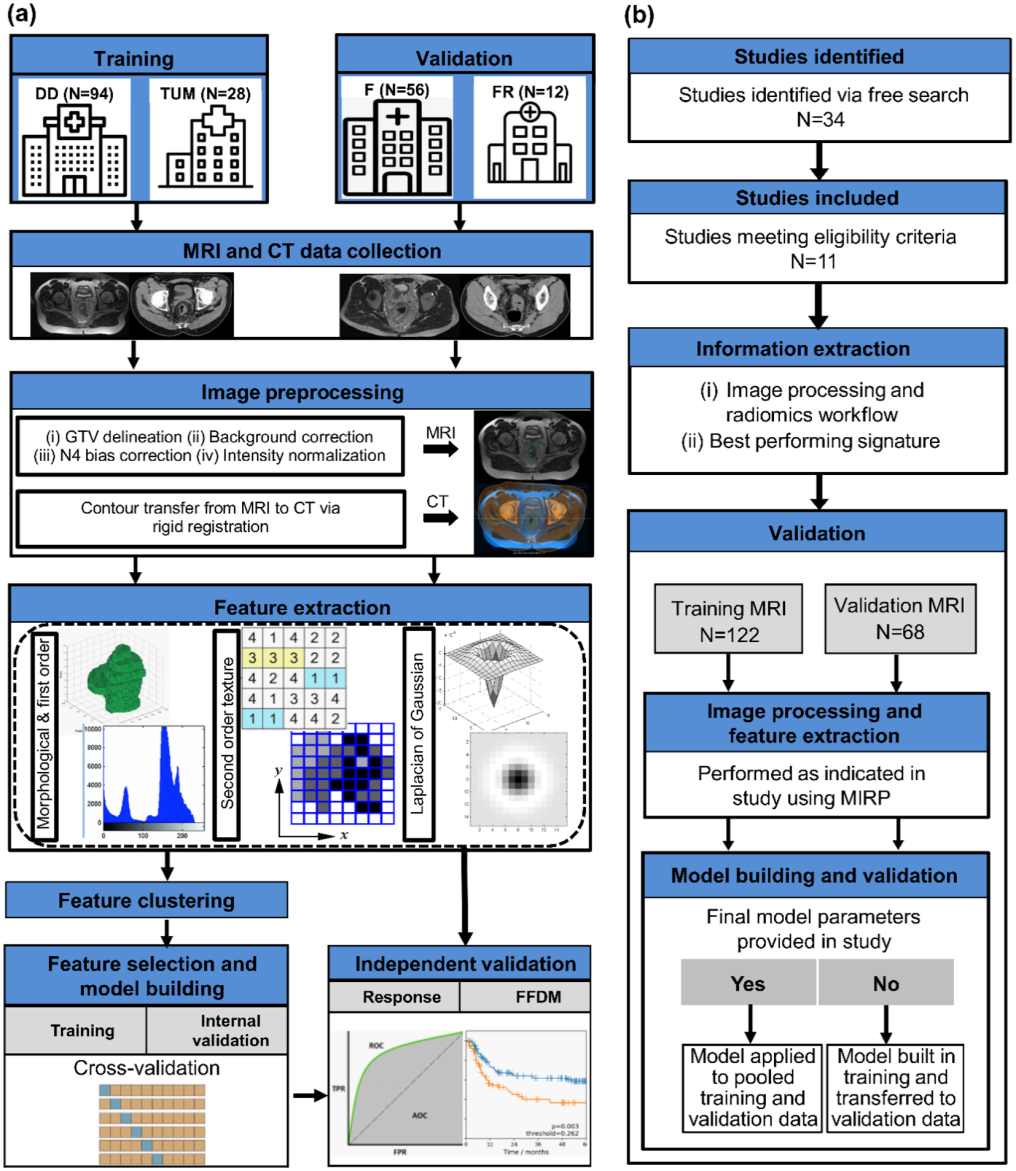
(**a**) Design of the modelling study. Treatment plan computed tomography (CT) and pre-treatment T2w magnetic resonance imaging (MRI) data were collected from 4 centres and divided into training and validation data. MRI data was preprocessed and gross tumour volume (GTV) was delineated, which was then transferred to CT images after rigid registration. Different feature classes were extracted from both modalities and signatures were developed on training data for tumour response prediction to neoadjuvant chemoradiotherapy (nCRT) and freedom from distant metastases (FFDM) in a cross-validation setting. These signatures were validated independently for both endpoints. (**b**) Design of the external validation study. Studies were identified via free search using Google scholar and PubMed and excluded if the inclusion criteria were not fulfilled. Information regarding image processing, radiomics workflow, and the best performing radiomics signature was extracted as reported. Image processing and feature extraction was reproduced using MIRP^34^. Finally, validation was performed either on the pooled training and validation data if model parameters were reported in the study or the model was re-trained on the training data and validated on the validation data.

In our modelling study, we developed and independently validated radiomic signatures for the prognosis of tumour response and FFDM in patients with LARC based on different radiomic feature classes. Figure 1a summarizes the design of this study. Imaging features were computed from the gross tumour volume (GTV) individually on the treatment-planning CT and pre-treatment T2w MRI, including morphological and first-order features (MFO), second-order texture features (SOT), and intensity features of Laplacian of Gaussian (LoG) transformed imaging. The features were filtered for stability under small image perturbations and clustered. In order to assess which image modality is more suitable for the prediction of the endpoints and which feature class has the highest prognostic value, four radiomic models were developed on the training data individually for each imaging modality based on (i) MFO, (ii) SOT, (iii) LoG, and (iv) all features, i.e. the combination of MFO, SOT, and LoG features. In an additional analysis, the selected features from CT and T2w MRI were combined for each of the cases (i) to (iv) to assess the benefit of multimodal radiomic models. The performance of each signature was then validated on the independent validation data using the area under the curve (AUC) and the concordance index (CI) for the prognosis of tumour response and FFDM, respectively. Details of image processing and modelling are described in the following paragraphs.

In our external validation study, we identified and validated radiomics biomarkers proposed for the prediction of tumour response to nCRT or FFDM from the literature (see Fig. 1b). A free search was carried out using google scholar and PubMed until October 2021.

The following free search keywords were used: ‘rectal cancer’ OR ‘Locally advanced rectal cancer’, ‘radiomics’, ‘response prediction’ OR ‘response to neoadjuvant chemoradiotherapy’, ‘distant metastases prediction’ OR ‘prognosis’, ‘deep learning’, ‘machine learning’. The studies were reviewed for eligibility based on the following criteria: (1) radiomics analysis on pre-treatment T2w MRI or CT without contrast agent, (2) radiomics features extracted from primary tumour (GTV), (3) normo-fractionated nCRT (dose 45–55 Gy) followed by surgery, (4) clear radiomics workflow and definition of finally used features available. The search and inclusion of studies were supervised by two reviewers (A.Z., S.L.). The following data were extracted from the included studies: (1) sample size and distribution to training and validation dataset (if any), (2) nature of study, i.e. single centre or multicentre, (3) clinical characteristics of patient cohort (4) used imaging modality, (5) reference standard for TRG, (6) image pre-processing workflow, (7) feature extraction geometry, i.e. 3D, 2D, or largest slice, (8) applied feature extraction framework, (9) final classification/regression model or statistical test, (10) features included in final model, (11) final model parameters (if any), and (12) reported results. The studies were arranged in chronological order of year of publication.

### Image acquisition

Imaging datasets were retrieved from the picture archiving and communication system (PACS) in the respective centres and pseudonymized centrally. Staging T2w MRI were acquired before nCRT with either a 1.5 T or a 3 T scanner. Patients received a CT scan for treatment planning prior to radiotherapy. Supplementary Table S2 summarizes MR and CT image acquisition and reconstruction parameters for training and validation data. The GTV was delineated for each patient on T2w transversal MR images by an experienced radiation oncologist and confirmed by a radiologist. CT images were coregistered with MRI using rigid registration in RayStation 8B SP2 (RaySearch Laboratories, Stockholm, Sweden) and the GTV was transferred to the CT.

### Image preprocessing, and feature extraction

Supplementary Figure S1 illustrates the process of image preprocessing used in the modelling study as previously described^26^. First, MR images were corrected for background phase variations that arise due to magnetic field inhomogeneities. This was achieved by creating a mask of the soft tissue region in the image using the Canny Edge detection algorithm and multiplying the true image with the mask, setting all the background phase variations to zero^32^. N4ITK bias correction method was used to minimize the bias field effect in MR images^33^. Image intensities were scaled using the 95th percentile of image intensities, i.e. 5% of the highest image intensities were ignored, representing potential outliers. Further image preprocessing followed by feature extraction was carried out using the MIRP Python toolkit (version 1.1.3)^34^. MR and CT image voxels were resampled to an isotropic voxel size of 1.0 × 1.0 × 1.0 mm^3^ using trilinear interpolation in order to adjust the voxel spacing and slice thickness between the datasets. In CT images, the GTV was re-segmented to cover only soft tissue voxels between − 150 and 180 Hounsfield units, removing voxels containing air and bone. A set of LoG filters with 5 different kernel widths (1 mm, 2 mm, 3 mm, 4 mm, 5 mm) was applied individually to the base MRI and CT images. The five response maps were averaged to a single image.

After image pre-processing, imaging features were computed. A set of 25 morphological and 57 first-order intensity-based features (MFO features) was extracted from the 3D GTV on the treatment planning CT and on the pre-treatment T2w MRI, respectively. In addition, 95 second-order texture features (SOT features) were calculated for every modality. Finally, the same 57 first-order intensity-based features were extracted from the GTV on the LoG transformed images. This resulted in a total of 234 features extracted from each imaging modality. SOT features were extracted from the 3D GTV based on the grey level co-occurrence matrix (GLCM), grey level run length matrix (GLRLM), grey level size zone matrix (GLSZM), grey level distance zone matrix (GLDZM), neighbourhood grey tone dependence matrix (NGTDM), and neighbouring grey level dependence matrix (NGLDM). Image pre-processing and feature extraction in MIRP were implemented according to the recommendations of the Image Biomarker Standardisation Initiative (IBSI)^35,36^. The definitions used to calculate the features can be found in the IBSI reference manual. Image processing parameters used for feature extraction are summarized in Supplementary Table S3.

In order to obtain reproducible results, imaging features have to be stable under small image perturbations, as e.g. caused by differing acquisition parameters or positioning uncertainties^37^. We evaluated feature robustness by applying the following image augmentations based on the training data: adding Gaussian noise (mean 0, standard deviation as present in the image), random volume changes of the GTV (0%, − 15%, 15%), and translations (0.0, 0.25, and 0.75 mm) in all three spatial dimensions. All combinations of these perturbations were considered, leading to 81 perturbed images for each original dataset. The intra-class correlation coefficient (ICC) was calculated with a 95% confidence interval, quantifying the similarity of feature values under different perturbations for every feature. Features with the lower boundary of the 95% confidence interval of the ICC below 0.8 were removed^37^.

The redundancy of features in MRI and CT was individually mitigated by hierarchical clustering, including (i) MFO features only, (ii) SOT features only, (iii) LoG features (statistical and intensity histogram) only, and (iv) all features, corresponding to the analyses based on the different feature classes. The Spearman correlation coefficient ($\rho$) was used as a similarity metric with average linkage as a criterion for merging two clusters; $\rho \ge$ 0.8 was defined for placing features into the same cluster. The feature with the highest mutual information with the endpoint was selected as the representative for each cluster.

For our external validation study, features were extracted from T2w MRI or CT data using MIRP. The features reported in each individual study were mapped to their closest synonyms in the IBSI manual. A feature was excluded from validation analysis if (i) it was not defined in the IBSI manual or (ii) MIRP cannot extract it. In that case, the remaining features were considered as candidates for validation. Image pre-processing (e.g. image interpolation, image normalization, bias correction) and feature extraction parameters (e.g. feature extraction in 2D, 3D or from the largest tumour area, discretization used for histogram or texture features, LoG or wavelet transformations) were replicated for each study if indicated. If feature extraction parameters were not mentioned in the study, the settings recommended in the IBSI standard were used.

### Radiomics modelling

In our modelling study, we evaluated 12 different radiomic models based on different (combinations of) feature classes and imaging modalities, as shown in Supplementary Fig. S2. First, four radiomic signatures were created individually for T2w MRI and CT based on (i) MFO, (ii) SOT, (iii) LoG, and (iv) all features. Once these signatures were developed, four signatures were created by joining the respective MRI and CT signatures from (i) to (iv).

In order to create the eight single-modality signatures, a workflow containing four major processing steps (Supplementary Fig. S2) was applied after feature clustering using an in-house end-to-end statistical learning software package: (i) feature preprocessing, (ii) feature-selection, (iii) model building with internal validation, and (iv) external validation. Steps (i)–(iii) were first performed using 33 repetitions of threefold stratified cross-validation (CV) nested in the training dataset to identify an optimal signature, i.e. the steps were repeatedly performed on the internal training part and validated on the internal validation part of the cross-validation folds. After identifying the final signature, a final model was developed on the entire training data and validated on the independent validation data.

The following procedure was performed for each of the 99 CV runs: (i) Features were transformed using the Yeo-Johnson transformation to align their distribution to a normal distribution. Afterwards, features were z-transformed to mean zero and standard deviation one. Both transformations were performed on the internal training part and the resulting transformation parameters were applied unchanged to the features of the internal validation part. (ii) Four supervised feature-selection algorithms were considered: minimal redundancy maximum relevance (MRMR)^38^, mutual information maximization (MIM)^39^, elastic-net^40^, and univariate regression. To avoid potential overfitting, only the five most relevant features were selected. (iii) The features selected by each of these methods were used to build prognostic models on the internal training part, which were validated on the internal validation part. Multivariable logistic regression was applied for the prognosis of tumour response and Cox regression for FFDM. Average model performance was assessed by the median cross validation AUC and CI for tumour response and FFDM prognosis, respectively, for every feature selection method.

After the cross-validation procedure, the final radiomic signature was created as follows. For each of the above-mentioned feature selection methods, the occurrence of every feature in the 99 modelling steps was counted and features were ranked according to their occurrence across the cross-validation folds. Features with occurrence ≥ 50% in at least 75% of the feature selection methods were selected and the cumulative occurrence of each feature was calculated as a sum of its occurrences. If a subset of these features showed a Spearman correlation $\rho$ > 0.5 on the entire training data, only the feature with the highest cumulative occurrence was considered. A detailed example of the feature selection scheme is presented in Supplementary Sect. 1, including Supplementary Tables S4–S6. The resulting radiomic signature was then used to build prognostic models on the entire training data and (iv) the trained model was applied to the independent validation data.

For creating the four joint signatures combining CT and MRI, the selected signatures in each feature class were pooled together and the same procedure as described in the last paragraph was performed: clusters with $\rho >0.5$ were reduced to one feature, models were trained on entire training data and validated on external validation data. Finally, clinical features that were significantly associated to tumour response in univariable logistic regression or to FFDM in univariable Cox regression were added to the selected radiomic signature (Supplementary Table S7).

In our external validation study, the pooled training and validation data was used for biomarker validation if a final model was provided in the respective study, or a statistical test was performed for associating the considered biomarker to the endpoint of interest. Otherwise, the given radiomic features were used to re-train a predictive model on the training data, which was subsequently validated on the validation data. Clinical features were combined with imaging biomarkers if mentioned in the study.

### Statistical analysis

The following baseline clinical parameters were available: gender, age, tumour localization, UICC stage, grading, T stage, N stage, surgery type, chemotherapy type. Categorical variables of the clinical data were compared between the training and validation data by the χ^2^ test whereas continuous variables were compared using the Mann–Whitney-*U* test.

Associations between the final model predictions and the endpoints were evaluated by the AUC for tumour response and by the concordance index (CI) for FFDM prognosis. The estimated value and the 95% confidence interval of these metrics were computed using the bias-corrected bootstrap confidence interval method on 400 bootstraps of the data^41^. For creating a confusion matrix based on the final signature for tumour response prediction, an optimal cutoff was selected on the training data using Youden index and transferred to the validation data. For association with FFDM, patients were stratified into an optimally separated low and a high-risk group using an optimal cutoff on the training data that was based on maximally selected rank statistics^42^. The cutoff was transferred to the validation data and FFDM of stratified groups was assessed with Kaplan Meier curves compared with the log-rank test.

Calibration for the prediction of tumour response to nCRT and FFDM was assessed via the Hosmer–Lemeshow goodness of fit test (HL test)^43^ and Greenwood Nam d’Agostino test (GND test)^44^, respectively. Correlations between features were assessed by the Spearman correlation coefficient ($\rho$). All tests were two-sided with a significance level of 0.05. The importance of individual features in the final signature was assessed by univariate fitting of a logistic regression (tumour response) or Cox regression (FFDM) and computing Wald-test p-values. All analyses were performed in R version 4.0.3.

## Results

### Modelling study: CT and MRI predict tumour response and FFDM

Patient characteristics of the training and validation data are summarised and compared in Table 1. Patients in the training data had a higher tumour grading (p = 0.001) and higher UICC stage (p < 0.001). Patients of the validation data were treated with a higher dose (p < 0.001). The endpoints tumour response and FFDM were similar for training and validation data (p = 0.13 and p = 0.25, respectively). In univariate analysis, a significant association was observed only between clinical T stage (cT) and tumour response (Supplementary Table S7).

**Table 1:** Patient, tumour, and treatment characteristics for the training and validation data.

Variable	Training data (122)		Validation data (68)		p-value
	Median	Range	Median	Range	
Age (years)	59.5	24–79	63.5	21–86	0.26
	**Number**	**%**	**Number**	**%**	
**Gender**					
Male/female	79/43	65/35	48/20	71/29	0.51
**cT**					
2/3/4/unknown	6/98/18/0	5/80/15/0	7/53/7/1	10/78/10/2	0.23
**cN**					
0/1/2/3/unknown	7/112/2/1/0	6/92/2/1/0	8/54/1/4/1	11/79/2/6/2	0.06
**Grading**					
0/1/2/3/unknown	10/5/71/36/0	8/4/58/30/0	4/3/53/5/3	6/4/78/8/4	0.001
**UICC stage**					
1/2/3/4/unknown	0/7/115/0/0	0/6/94/0/0	1/7/52/3/5	2/10/77/4/7	< 0.001
**Localization (cm)**					
3–6/> 6–12/> 12–16	65/54/3/0	53/44/3/0	24/37/6/1	35/54/9/2	0.02
**RT dose (Gy)**					
50.4/45	95/27	78/22	66/2	97/3	< 0.001
**Chemotherapy regimen**					
5FU/5FU + OX/CAP/CAP + other	97/10/7/8	80/8/6/7	59/7/2/0	87/10/3/0	0.13
**Response (TRG)**					
0/1/2/3/4	0/23/61/24/14	0/19/50/20/11	3/14/30/10/11	4/21/44/15/16	0.13
**Distant metastases**					
No/yes	103/19	84/16	52/16	76/24	0.25

*cT* clinical T stage, *cN* clinical N stage, *RT* radiation therapy, *TRG* tumour regression grade, *CAP* capecitabine, *OX* oxaliplatine, *FU* fluorouracil.

For radiomics modelling, 234 radiomic features were extracted from the GTV in the T2w MR and in the CT imaging dataset. Stability analysis reduced these to 208 features (MFO: 74, SOT: 82, LoG: 52) and 222 (MFO: 76, SOT: 95, LoG: 51) for MRI and CT, respectively. Clustering of correlated features further reduced the feature number to (i) MRI_MFO_:24, CT_MFO_:22; (ii) MRI_SOT_:16, CT_SOT_:19; (iii) MRI_LoG_:14, CT_LoG_:15; and (iv) MRI_All_:39, CT_All_:47.

Table 2 presents the results for the prognosis of tumour response, including the names of finally selected features. In internal cross validation, models based on CT data showed better prognostic performance than models based on MRI. Among feature classes, SOT features showed a high prognostic value (MRI: AUC_SOT_ = 0.68, AUC_MFO_ = 0.57, AUC_LoG_ = 0.57, AUC_All_ = 0.65; CT: AUC_SOT_ = 0.70, AUC_MFO_ = 0.65, AUC_LoG_ = 0.64, AUC_All_ = 0.67). This result, however, did not translate to the independent validation data, where SOT features performed poorly. Here, the overall best performance was achieved by LoG features for both imaging modalities (MRI: AUC_LoG_ = 0.66, CT: AUC_LoG_ = 0.61). Joint MRI + CT signatures performed almost similar to MRI only signatures in independent validation for all four models.

**Table 2:** Median area under the curve (AUC) values for cross validation (CV) and external validation for tumour response prediction based on MRI, CT, joint MRI + CT, and imaging combined with clinical T stage. Values in parenthesis represent the 95% confidence interval.

Modality		Feature level	CV training AUC	CV validation AUC	Signature	Final training AUC	External validation AUC
MRI		All	0.76	0.65	MR_dzm_zd_entr_3d_fbn_n32	0.72 (0.62–0.82)	0.34 (0.19–0.50)
		MFO	0.74	0.57	MR_morph_av MR_morph_geary_c	0.70 (0.60–0.79)	0.57 (0.39–0.73)
		SOT	0.75	0.68	MR_dzm_zd_entr_3d_fbn_n32	0.72 (0.62–0.81)	0.34 (0.10–0.50)
		LoG	0.70	0.57	MR_log_ih_max_grad_fbn_n32 MR_log_stat_min	0.67 (0.57–0.75)	0.66 (0.51–0.82)
CT		All	0.78	0.67	CT_dzm_zd_var_3d_fbn_n32 CT_cm_corr_d1_3d_v_mrg_fbn_n32	0.77 (0.69–0.84)	0.47 (0.34–0.63)
		MFO	0.77	0.65	CT_morph_av	0.72 (0.60–0.82)	0.52 (0.38–0.66)
		SOT	0.78	0.70	CT_dzm_zd_var_3d_fbn_n32 CT_cm_corr_d1_3d_v_mrg_fbn_n32	0.77 (0.59–0.80)	0.47 (0.36–0.66)
		LoG	0.73	0.64	CT_log_ih_max_grad_fbn_n32	0.70 (0.60–0.79)	0.61 (0.44–0.76)
Joint MRI + CT		MRI_All + CT_All			MR_dzm_zd_entr_3d_fbn_n32 CT_cm_corr_d1_3d_v_mrg_fbn_n32	0.76 (0.67–0.84)	0.38 (0.24–0.56)
		MRI_MFO + CT_MFO	–	–	MR_morph_geary_c CT_morph_av	0.74 (0.64–0.83)	0.57 (0.40–0.67)
		MRI_SOT + CT_SOT	–	–	MR_dzm_zd_entr_3d_fbn_n32 CT_cm_corr_d1_3d_v_mrg_fbn_n32	0.76 (0.67–0.84)	0.38 (0.24–0.56)
		MRI_LoG + CT_LoG	–	–	MR_log_stat_min CT_log_ih_max_grad_fbn_n32	0.71 (0.62–0.80)	0.66 (0.50–0.82)
Clinical + MRI/CT		No Radiomics	–	–	cT	0.60 (0.53–0.66)	0.60 (0.50–0.70)
		MRI_LoG	–	–	cT MR_log_ih_max_grad_fbn_n32 MR_log_stat_min	0.69 (0.59–0.78)	0.69 (0.53–0.82)
		CT_LoG			cT CT_log_ih_max_grad_fbn_n32	0.72 (0.61–0.81)	0.66 (0.51–0.81)
		MRI_LoG + CT_LoG	–	–	cT MR_log_stat_min CT_log_ih_max_grad_fbn_n32	0.72 (0.62–0.80)	0.70 (0.54–0.84)

*AUC* area under a curve, *cT* clinical T stage, *CT* computed tomography, *CV* cross-validation, *LOG* Laplacian of Gaussian, *MRI* magnetic resonance imaging, *MFO* morphological and first order, *SOT* second order texture.

**Figure 2:**
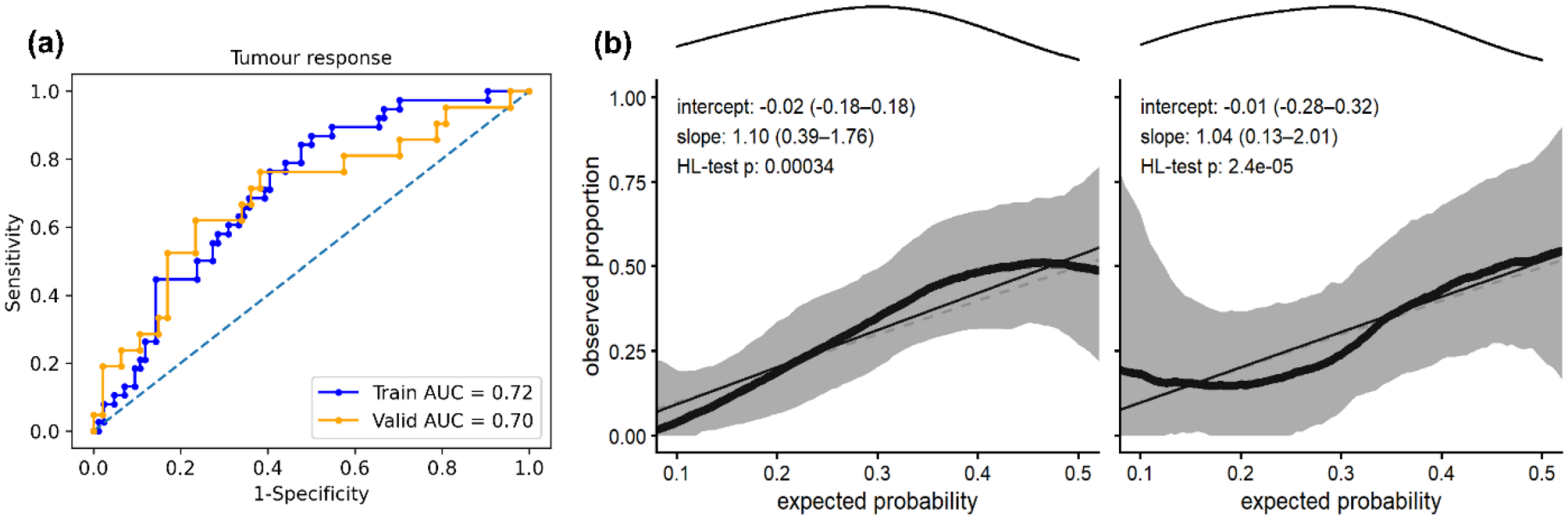
(**a**) Receiver operating characteristics (ROC) curves and (**b**) calibration plots for tumour response prognosis in training (left) and validation (right) resulting from best performing joint signature combining clinical T stage and Laplacian of Gaussian (LoG) features from T2w-MRI and CT. For calibration, data (thick lines) and 95% confidence intervals (shaded regions) are shown together with linear regression lines (solid lines) that follow the optimal expectation (dashed lines). Density of expected probabilities is shown above the calibration plot.

**Figure 3:**
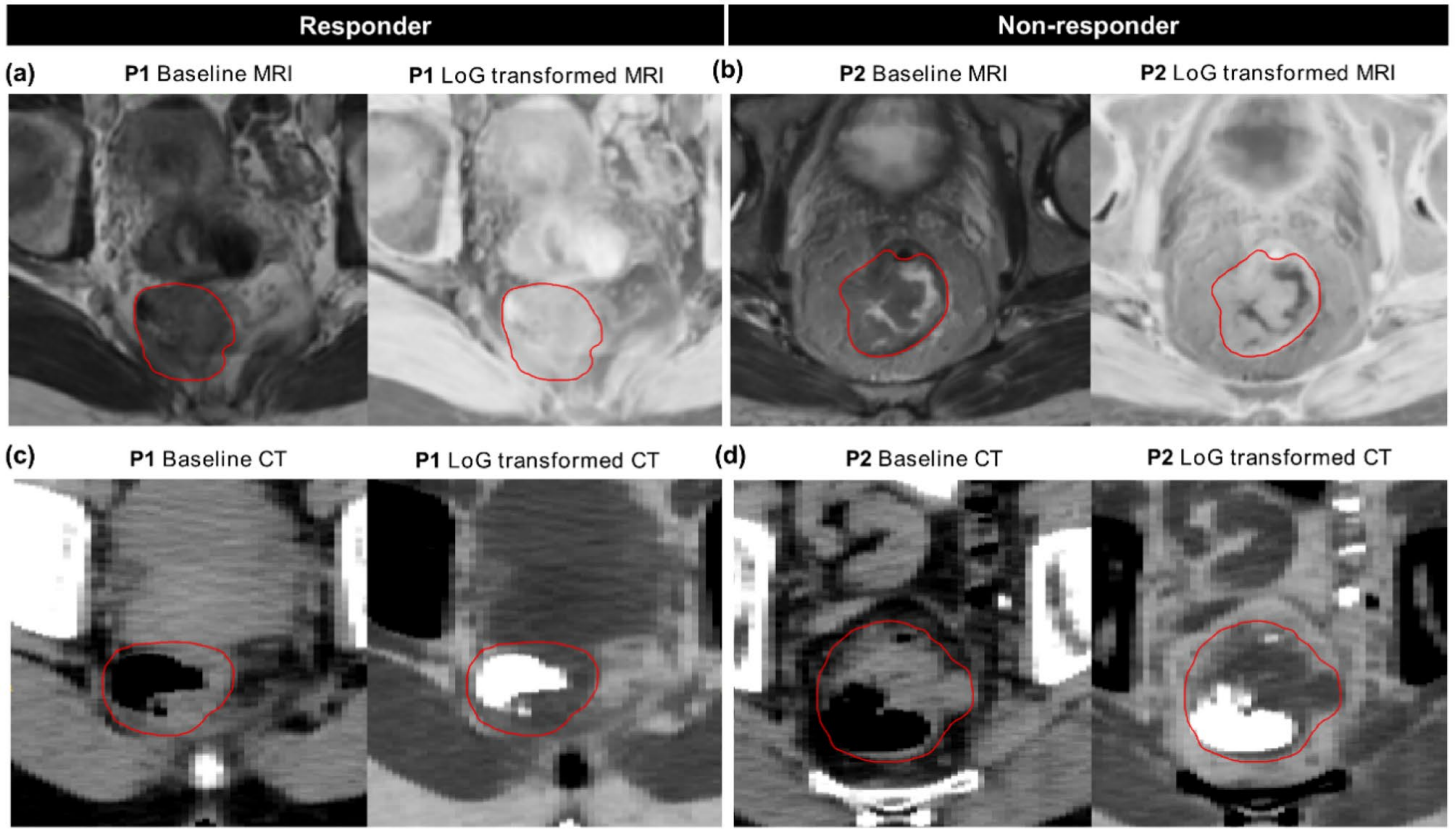
Representative images from MRI (**a**,**b**) and CT (**c**,**d**) with corresponding Laplacian of Gaussian (LoG) transformed images from two patients (P) in the two response groups, i.e. responder: P1 and non-responder: P2 on the training data. Red contours mark the gross tumour volume (GTV). P1 (responder: TRG = 4) showed an overall homogenous appearance on the baseline MRI. On the contrary, P2 (non-responder: TRG = 1) showed a more heterogeneous GTV with a low stat_min value on the LoG transformed MR image, which corresponds to some high pixel intensities on the baseline MRI. Similarly, a more homogenous GTV (excluding the air voxels) can be seen in P1 compared to P2 on the baseline and LoG transformed CT slices, possibly causing low gradients in the intensity histogram for the responder.

The clinical model containing only cT stage achieved training and validation AUCs of 0.60. Combining cT stage with the combined signature from MRI and CT achieved the best validation result with an AUC of 0.70. At a threshold of 0.248 this signature was able to accurately classify 16/21 responders and 20/47 non-responders (Supplementary Fig. S3). Figure 2 shows receiver operating characteristic (ROC) curves and the corresponding calibration plots for this signature on training and validation data. All features represented independent information (Supplementary Fig. S4) and significantly contributed to the prediction in training (p < 0.05), while only MR_log_stat_min was significant in validation (p = 0.04). The MRI feature log_stat_min (IBSI:1GSF) represents the minimum intensity, while the CT feature log_ih_max_grad_fbn_n32 (IBSI:12CE) represents the gradient of the discretised histogram (32 bins) within the GTV on the LoG transformed image. Image-based interpretation of these features is presented in Fig. 3. In the non-responder group, MR_log_stat_min showed relatively low values, which translates to the existence of bright voxels in the GTV on the original baseline T2w MRI (Fig. 3b). In comparison, responders showed no such high grey values (Fig. 3a). Box plots of these features (Yeo-Johnson transformed and z-score normalized) in the two response groups are shown in Supplementary Fig. S5.

**Figure 4:**
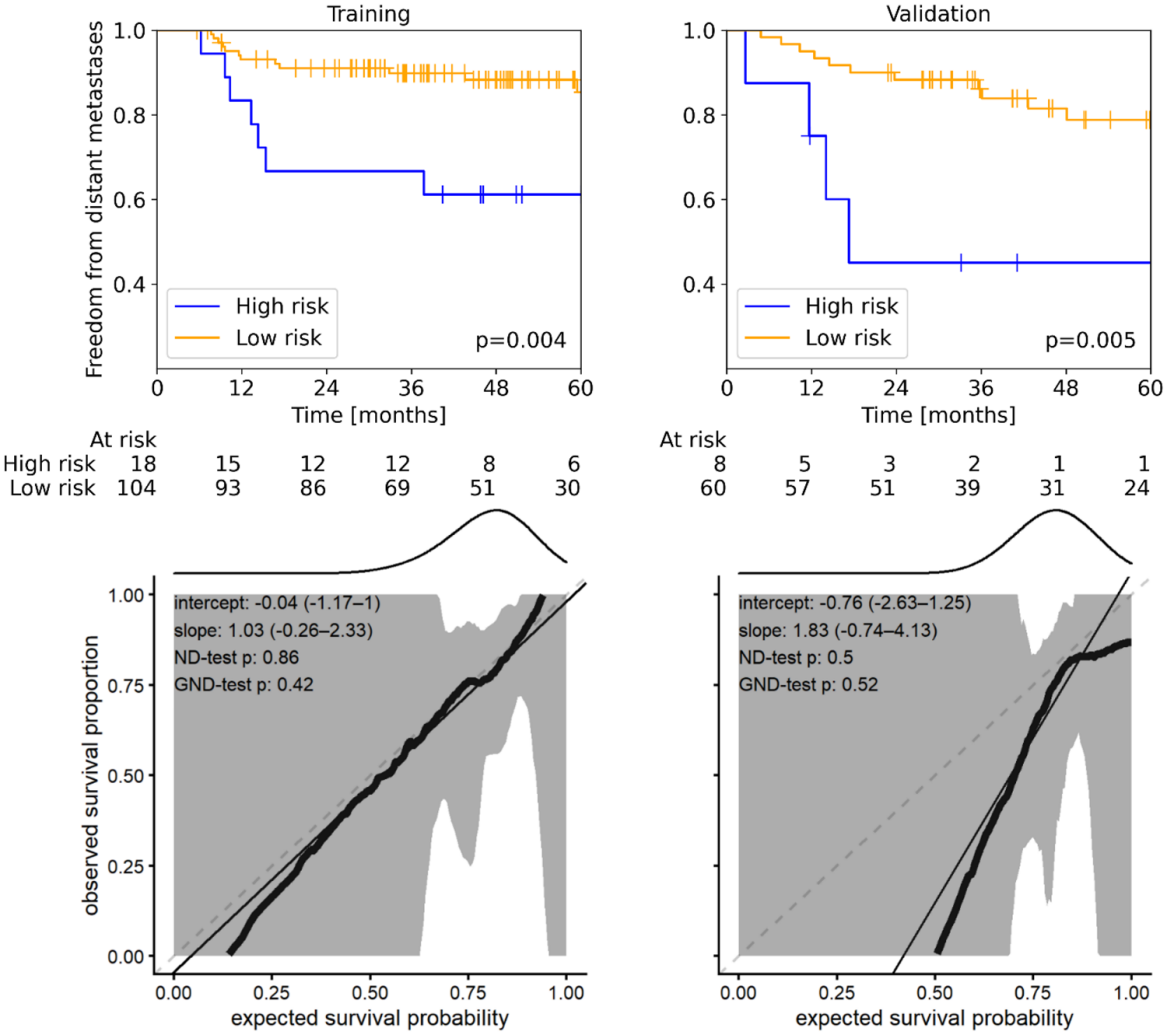
Kaplan–Meier (top) and calibration plots (bottom) on training (left) and validation (right) data for the prediction of FFDM using the three best performing CT-based SOT features, resulting in significant patient stratifications (p < 0.01). For calibration, data (thick lines) and 95% confidence intervals (shaded regions) are shown together with linear regression lines (solid lines) that should follow the optimal expectation (dashed lines). Density of expected probabilities is shown above the calibration plot.

Table 3 presents the results for the prognosis of FFDM, including the names of finally selected features. Median follow up time in training and validation data was 49.1 (5.7–111.8) months and 29.5 (1.2–94.1) months, respectively. Most of the metastases occurred until 24 months after treatment (training: 76%, validation: 56%). Until that time, 7 patients (training: 5 validation: 2) were lost to follow-up because of death, i.e. the competing risk of death was small. In internal cross validation, models based on MRI data showed a better prognostic performance than models based on CT. Among feature classes, LoG features showed a somewhat higher prognostic value (MRI: CI_LOG_ = 0.65, CI_MFO_ = 0.60, CI_SOT_ = 0.59, CI_All_ = 0.60, CT: CI_LOG_ = 0.52, CI_MFO_ = 0.47, CI_SOT_ = 0.51, CI_All_ = 0.46). In external validation, CT-based features showed a slightly higher performance compared to MRI. While both SOT and LoG features achieved similar prognostic value on MRI data (MRI: CI_SOT_ = 0.57, CT: CI_LoG_ = 0.57), the overall best prognostic performance in CT was achieved by SOT features (CT: CI_SOT_ = 0.69). No additional benefit was achieved by joining the MRI and CT signatures. Patient stratification into groups at low and high risk of distant metastases was performed based on the SOT models for each modality, i.e. for MRI, CT, and joint MRI + CT. While the CT and MRI + CT-based signatures achieved a significant patient stratification in independent validation (p < 0.01), this was not the case for the MRI-based signature (p = 0.68). Kaplan–Meier curves and corresponding calibration plots for the best performing CT signature are shown in Fig. 4 and for the MRI and MRI + CT signatures in Supplementary Fig. S6. The definition and interpretation of selected features with corresponding optimal thresholds for patient stratification are presented in Supplementary Table S8.

**Table 3:** Median concordance index (CI) values for cross-validation (CV) and external validation for FFDM prediction in MRI, CT, and joint MRI + CT. Values in parenthesis represent the 95% confidence interval.

Modality	Feature level	CV training CI	CV validation CI	Signature	Final training CI	External validation CI
MRI	All	0.79	0.60	MR_log_stat_median	0.69 (0.56–0.81)	0.54 (0.36–0.69)
	MFO	0.77	0.60	MR_stat_median	0.68 (0.54–0.82)	0.52 (0.34–0.68)
	SOT	0.75	0.59	MR_ ngl_dc_var_d1_a0_0_3d_fbn_n32 MR_ szm_sze_3d_fbn_n32 MR_cm_clust_prom_d1_3d_v_mrg_fbn_n32	0.70 (0.58–0.82)	0.57 (0.40–0.74)
	LoG	0.75	0.65	MR_log_stat_median MR_log_stat_iqr MR_log_ih_entropy_fbn_n32	0.69 (0.56–0.82)	0.57 (0.39–0.73)
CT	All	0.74	0.46	No feature selected	–	–
	MFO	0.73	0.47	CT_morph_volume	0.62 (0.50–0.75)	0.58 (0.42–0.73)
	SOT	0.70	0.51	CT_szm_zsnu_3d_fbn_n32	0.64 (0.49–0.80)	0.69 (0.51–0.81)
	LoG	0.70	0.52	CT_log_stat_energy	0.65 (0.53–0.76)	0.63 (0.46–0.77)
Joint MRI + CT	MRI_All + CT_All	–	–	MR_log_stat_median	0.69 [0.56–0.81]	0.54 (0.36–0.69)
	MRI_MFO + CT_MFO	–	–	MR_stat_median CT_morph_volume	0.70 [0.55–0.81]	0.55 (0.37–0.70)
	MRI_SOT + CT_SOT	–	–	MR_ ngl_dc_var_d1_a0_0_3d_fbn_n32 MR_ szm_sze_3d_fbn_n32 MR_cm_clust_prom_d1_3d_v_mrg_fbn_n32 CT_szm_zsnu_3d_fbn_n32	0.73 (0.61–0.84)	0.62 (0.45–0.79)
	MRI_LoG + CT_LoG	–	–	MR_log_stat_median MR_log_stat_iqr MR_log_ih_entropy_fbn_n32 CT_log_stat_energy	0.72 (0.59–0.85)	0.59 (0.41–0.75)

*CI* concordance-index, *CT* computed tomography, *CV* cross-validation, *LOG* Laplacian of Gaussian, *MRI* magnetic resonance imaging, *MFO* morphological and first order, *SOT* second order texture.

Supplementary Table S9 contains model and transformation parameters for the best performing signatures developed for tumour response and FFDM prediction. Training was performed on the entire training data.

### External validation study: most previous studies could not be validated

In total, 34 studies were identified as relevant based on their titles and abstracts. All identified studies were performed on patients with LARC that were treated with nCRT followed by surgery with the aim of predicting tumour response using radiomics. 23 studies were excluded after full text review due to following reasons: 3 studies used contrast enhanced CT data that was not available in our dataset^21,45,46^, 4 studies used both pre and\or post treatment data^47–50^, 5 studies used pre-treatment multiparametric MRI (mpMRI) to develop a final signature with no standalone T2w MRI signature being reported^17,18,51–53^, 2 studies did not report any final signature^22,30^, 3 studies could not be reproduced as the radiomics workflow or feature definition was not clearly explained^25,54,55^, 1 study was excluded as the considered ROI was not the primarytumour^56^, 3 studies were excluded as authors reported failure of radiomics to predict the outcome of interest^57–59^, 2 studies were excluded as the reported signature was computed from feature maps, which are currently not supported by MIRP^28,60^. Finally, eleven studies were included for external validation analysis. All of them used T2w MRI for predicting tumour response and were published between 2015 and 2020. One study was prospective, nine were retrospective, and three were multicentric. Two of these multicentre studies considered clinical features and imaging biomarkers.

Our external validation results are summarized in Table 4. The considered biomarkers and their corresponding synonyms together with image processing and feature extraction details for included studies are summarized in Supplementary Appendix 2 and Supplementary Table S10, clinical characteristics of the studies are given in Supplementary Table S11. Except for one study, none of the included studies could be validated, i.e. they showed p-values above 0.05 and/or a training/validation AUC significantly below the reported value in the study with a 95% confidence interval including the value 0.5. The only study that could be validated is by Petkovska et al.^14^. An acceptable performance was observed on our pooled data (AUC = 0.64 [0.51–0.77]). Supplementary Figure S7 shows the calibration plot for this study. In a study by Chidbaram et al.^27^, pathological complete responders showed a significant association with tumour volume delineated on T2w image (Mann–Whitney-U test p = 0.013). This was somewhat confirmed in our analysis, where we observed a statistical trend (p = 0.061). However, radiomics analyses are not needed to assess the tumour volume. For the study by Antunes et al.^11^, the random forest (RF) model created on a single feature was not successful on our training data but achieved an acceptable performance on the validation data (AUC: Train, Validation = 0.48, 0.63). Still, on the pooled training and validation data the selected feature was insignificant (Mann–Whitney-U test p = 0.12).

**Table 4:** Overview of studies included in validation analysis. For all included studies, patients were treated with nCRT followed by resection. Radiomics analysis was reported on pre-treatment T2w MRI with features extracted from the primary tumour region. The column validation approach indicates whether model coefficients or statistical tests were applied on the pooled training and validation data (Pooled) or the model was re-trained on the training data and validated on the validation data (train/valid). *AUC* area under a curve (with 95% confidence interval in brackets), *MRI* magnetic resonance imaging, *nCRT* neoadjuvant chemoradiotherapy.

Study	Study type	Validation approach	Final results from study	Results from validation analysis (unadjusted p-value)
De Cecco (2015, 2016)^16,65^	Prospective, single centre	Pooled	AUC = 0.91, 0.86 p-value = 0.01, 0.01	AUC = 0.56 (0.44–0.68) p-value = 0.31
Chidbaram (2017)^27^	Retrospective, single centre	Pooled	p-value = 0.013	p-value = 0.061
Caruso (2018)^13^	Retrospective, single centre	Pooled	p-values < 0.05 for all features	p-values > 0.05 for all features
Casumano (2018)^12^	Retrospective, multicentre	Pooled	AUC = 0.79	AUC = 0.58 (0.46–0.70)
Dinapoli (2018)^10^	Retrospective, multicentre	Pooled	AUC = 0.75	AUC = 0.59 (0.47–0.71)
Meng (2018)^66^	Retrospective, single centre	Pooled	p-value = 0.02	p-value = 0.098
Cui (2019)^67^	Retrospective, single centre	Pooled	AUC = 0.73	AUC = 0.52 (0.38–0.64)
Antunes (2020)^11^	Retrospective, multicentre	Train/valid	Train\Valid AUC = 0.699\0.712 Skewness-Laws Wave-Ripple (p-value Train = 1.6 × 10 − 4)	Results on Skewness-Laws Wave-Ripple Train\valid AUC = 0.48 (0.36–0.57)\0.63 (0.52–0.76) p-value Train\valid = 0.71\0.055 p-value Pooled = 0.12
Petkvoska (2020)^14^	Retrospective, single centre	Pooled	AUC = 0.75	AUC = 0.64 (0.51–0.77)
Petresc (2020)^15^	Retrospective, single centre	Pooled	AUC = 0.80	AUC = 0.48 (0.38–0.57)

## Discussion

In this study, we developed and validated radiomics signatures incorporating pre-treatment T2w MRI and treatment planning CT imaging features for the prediction of tumour response to nCRT and FFDM in patients with LARC. The discriminative performance of MFO, SOT, LoG, and combination of all features was independently validated for each imaging modality and their combination. Clinical T stage combined with LoG features from CT and MRI showed the best validation performance for the prediction of tumour response (AUC = 0.70), while SOT features from CT showed best performance for FFDM (CI = 0.69). Furthermore, we aimed to externally validate previously published radiomics signatures developed for tumour response prediction based on our multicentre data. Remarkably, no significant results were obtained, except for one study by Petkovoska et al.^14^ (AUC = 0.64), which overall indicates a potential lack of reproducibility for radiomics studies (see below).

Considering MRI-based multicentre radiomic studies with an independent validation for patients with LARC, the prognostic performance of our best performing signature (AUC = 0.70) was similar to the results of Antunes et al.^11^ (AUC = 0.71), but lower than results presented by Cusumano et al.^12^ (AUC = 0.79) and Dinapoli et al.^10^ (AUC = 0.75), who also assessed tumour response to nCRT in LARC patients using T2w MRI data. Antunes et al.^11^ used features extracted from laws kernels and gradient organization responses. In our validation analysis, only skewness-laws features could be validated. The corresponding feature used by Antunes et al.^11^ was not significant in training and showed a statistical trend in validation (p = 0.055). Dinapoli et al.^12^ used first-order intensity histogram-based features, while the study by Cusumano et al.^12^ additionally used fractal features in the final signature to build the model. Both studies also combined clinical features (cT and cN) with the radiomics signature. In our validation study, these signatures did not show a good performance (AUC < 0.60).

Single centre retrospective studies have also shown promising results for tumour response prediction in LARC. De Cecco et al.^16^ and Caruso et al.^13^ showed a significant association (p < 0.05) of FO statistical and GLCM features, respectively, with tumour response to nCRT on small cohorts (≤ 15 subjects). However, in our validation analysis, no significant association has been found for these features (p > 0.05). Coppola et al.^28^ showed that heterogeneity of local skewness is associated to tumour response (AUC = 0.90). Ferrari et al.^60^ showed that complete responders have higher GLCM energy and good responders have high expression of histogram features (AUC = 0.87). These studies could not be validated as the features were extracted from feature maps, which are currently not supported in MIRP. More recent studies showed the association of SOT features with tumour response prediction. The studies by Pizzi et al.^30^ and Petresc et al.^15^ showed an AUC of 0.79 and 0.80 in internal validation, respectively. However, validating the results of Petresc et al.^15^ on our multicentre data was not successful (AUC = 0.48).

Fewer studies have investigated the performance of CT imaging for tumour response prediction to nCRT using patient populations treated with standard procedures, i.e. nCRT followed by TME^21,22,57,59^, or combined CT and MR imaging^25,61^. Bibault et al.^22^ developed a model for the prognosis of tumour response with radiomics features extracted from treatment plan CT data using deep neural networks (DNN) with an AUC of 0.72. Chee et al.^21^ demonstrated that pre-treatment contrast enhanced CT-based FO features were associated with tumour response prediction (responders showed low entropy, high uniformity, and low standard deviation). Other studies indicated an overall poor performance of CT features for predicting tumour response. Exemplarily, Rao et al.^59^ and Hamerla et al.^57^ showed that CT features were not able to predict tumour response. Regarding the combination of CT and MRI, Zhang et al.^61^ used MFO and SOT features extracted from pre-treatment CT and MRI and achieved an AUC of 0.87, while Li et al.^25^ showed that contrast enhanced CT and multimodality MRI is able to achieve an AUC of 0.93. While these studies showed promising results, they mostly lacked external validation.

Model performance may be improved by including additional imaging time points, other MRI sequences, or PET. Exemplarily, Jeon et al.^28^ used delta-radiomic features extracted from pre- and post-nCRT T2w MRI to build predictive signatures for treatment outcomes in LARC. Their signature showed significant risk group stratification for FFDM (p < 0.05). Chiloiro et al.^62^ also used delta radiomics to predict FFDM as binary outcome with an AUC of 0.78. To the best of our knowledge, no study was yet performed to predict FFDM combining pre-treatment MRI and treatment-planning CT for LARC. Gianni et al.^29^ showed that radiomic signatures based on PET, T1w MRI, and apparent diffusion coefficient (ADC) images had an increased performance for tumour response prediction (AUC = 0.86) compared to PET only (AUC = 0.84) and T1w MRI only (AUC = 0.72).

In radiomics analyses, numerous features of different complexity can be extracted and frequently their number is larger than the study population, which can lead to substantial model overfitting and difficult feature interpretability. In internal cross-validation, we observed that more complex SOT features showed a high performance for tumour response prediction, while LoG transformed intensity features showed a high performance for the prediction of FFDM. However, in external validation, the opposite behaviour was observed, i.e. LoG transformed statistical and intensity histogram features showed a high performance for the prediction of tumour response, while SOT features showed a somewhat higher performance for FFDM prediction. Also, it is noteworthy that the performance trend of feature classes in internal and external validation was similar for both modalities, i.e. similar feature classes were predictive for both CT and MRI. Specifically, we discovered one MRI-based statistical feature, i.e. log_stat_min, which was predictive for tumour response to nCRT. This feature represents the minimum intensity on LoG transformed images, which is closely related to the maximum intensity (i.e. stat_max) on baseline images. We analysed the predictive performance of both features separately using univariate logistic regression. In training, stat_max was less predictive (AUC = 0.57) then log_stat_min (AUC = 0.64), while both features showed similar performance in validation with an AUC of 0.66. The high association of LoG transformed intensity features with the training data can be attributed to the fact that the LoG kernels help to reduce large variations in the signal, which can be detected within a single image slice (e.g. irregularities due to magnetic field, respiratory motion, or patient movement). Further, we interpret log_stat_min as a potential biomarker for tumour response prediction to nCRT based on the fact that a tumour normally is represented by low to intermediate signal intensity on T2w MRI, excluding the intestinal lumen^48,63^. The increased expression of log_stat_min in non-responders indicates the presence of high intensities within the GTV on baseline T2w MRI, possibly indicating an aggressive or resistive tumour resulting in incomplete remission.

One major issue in radiomics analyses is feature reproducibility and the lack of consensual guidelines on which features have to be extracted from clinical imaging data. In our validation study, we experienced limited reproducibility of published literature. Only 32% of the eligible literature could be assessed for their validation performance with our data/methods, mostly due to the use of different software implementations and underreporting of methods employed for radiomics analysis of LARC. Important details such as image processing for feature extraction (e.g. discretization for intensity and texture features), final signatures together with their interpretation and final models were not always provided. Thus, there is a strong need of standard radiomics process for signature definition for both reproducibility and progression of radiomics towards clinical application. The IBSI^35^ aims to establish such a consensus and reporting guidelines for image processing and feature extraction. Although some studies have used large cohorts for radiomics analyses in LARC, external validation was rarely performed. Only 4 studies^10–12,56^ have used retrospective multicentre cohorts with a maximum of 3 data centres involved, which may lead to a low generalizability of the presented radiomic signatures. To tackle such problems, in our multicentre study, we have established and externally validated radiomics signatures in accordance with the IBSI guidelines and we report parameters and algorithms used for their extraction, transformation, stability analysis, and modelling.

In addition to the lack of standardization in the radiomics workflow, there is lack of standardized imaging protocols as well. This can obstruct the successful validation of radiomics models, e.g. for imaging from MR scanners of different vendors or different magnetic field strengths, because such differences may lead to the extraction of differently distributed features^64^. Standardization at hardware level is costly, thus there is a need to develop generalizable models by incorporating data from different scanners and protocols. We addressed this issue by using multicentre data independent of vendor and imaging protocols for training and validation. Furthermore, we observed significant differences between the clinical characteristics of our pooled cohort and the external cohorts included in the validation study (mainly clinical T and N stage). These differences may explain part of the observed reduced performance of the published models in our external validation analysis.

Limitations of this study are its retrospective nature and the relatively low number of patients in the training and validation data. In addition, there is a class imbalance due the smaller number of events for both endpoints, leading to wide confidence intervals in Tables 2 and 3 often including the value 0.5, i.e., the external validation results have a relatively large uncertainty. We aimed to mitigate this problem by internal cross-validation (CV) on the training data for feature selection. A threefold CV approach was used and repeated 33 times, ensuring that each fold contained sufficient events for training and validation and that the finally considered average model performance was sufficiently robust. A common strategy used in machine learning to tackle the problem of imbalanced data is random undersampling of the majority class. We tested this procedure during stratified splitting of training data in internal cross-validation. We did not observe significant differences in feature selection for both endpoints and therefore do not present the results from these experiments.

In conclusion, in the present modelling study, we developed and independently validated radiomic signatures for the prognosis of tumour response to nCRT and FFDM in patients based on T2w MR and CT imaging. We studied feature classes of differing complexity and observed that a combination of LoG transformed intensity features from MRI and CT together with clinical T stage (cT) led to the highest prognostic value for the prediction of nCRT, while CT-based SOT features performed well in external validation for FFDM. In our external validation study, only one of the radiomics signatures could be validated. This indicates an overall lack of reproducibility and the need for standardization in radiomics procedure and reporting before its prospective clinical application.

## Supplementary Information

Supplementary Information.

## Data Availability

The data that support the findings of this study are available on request from the corresponding author (S.L.). The data is not publicly available due to patient data privacy policy.
