# Supplementary material for: Analysis of MRI and CT-based radiomics features for personalized treatment in locally advanced rectal cancer and external validation of published radiomics models
Source: Sci Rep. 2022 Jun 17;12:10192. doi: 10.1038/s41598-022-13967-8 (PMC9205935; doi:10.1038/s41598-022-13967-8)
Supplement: Supplementary file 1 — Supplementary Information. [file 41598_2022_13967_MOESM1_ESM.pdf]

Supplementary Material: Analysis of MRI and CT-based radiomics features for personalized treatment in locally advanced rectal cancer and external validation of published radiomics models

Iram Shahzadi; Alex Zwanenburg; Annika Lattermann; Annett Linge; Christian Baldus; Jan C. Peeken; Stephanie E. Combs; Markus Diefenhardt; Claus Rödel; Simon Kirste; Anca-Ligia Grosu; Michael Baumann; Mechthild Krause; Esther G.C. Troost and Steffen Löck

Table S1: Tumour regression grade (TRG) system following Dowark et al [1].

Grade	Description
Complete regression (TRG 4)	No tumour cells
Near complete regression (TRG 3)	Very few tumour cells
Moderate regression (TRG 2)	Dominantly fibrotic changes with few tumour cells or groups
Minimal regression (TRG 1)	Dominant tumour mass with obvious fibrosis
No regression (TRG 0)	No regression

Table S2: Image acquisition parameters of diagnostic T2w magnetic resonance imaging (MRI) and treatment planning computed tomography (CT) data.

MRI			CT		
**Imaging parameters**	**Training (122)**	**Validation (68)**	**Imaging parameters**	**Training (122)**	**Validation (68)**
**Voxel spacing / mm**			**Voxel spacing / mm**	119/2/0/1	57/3/8
1.0/0.9/0.8/0.7/0.6/0.5/0.4/0.3	1/5/33/50/27/4/1/1	4/1/15/17/17/9/1/4	1/0.9/0.8/0.7		
**Slice thickness / mm**			**Slice thickness / mm**		
7/6/5/4/3	0/4/53/35/30	1/5/8/3/51	3/5	70/52	56/12
**Flip angle / °**			**Reconstruction kernel**		
180/160/150/141-147/132-137/120-127/90	69/1/22/2/4/6/18	4/15/36/1/2/2/8	B/B20f/B30f/B31s/B40s/B41s/59.10.AB50/unknown	0/1/0/23/86 0/3/9	56/0/1/0/0/11/0/0
**Scanning sequence**			**Exposure time / ms**		
SE/RM	96/26	68/0	500/800/1000/1200/1500/unknown	1/3/86/0/23/9	1/0/0/1/11/55
**Field strength / T**			**Tube voltage / kV**	4/109/0/9	56/11/1/0
1.5/3	103/19	51/17	120/130/140/unknown		
**Manufacturer**			**Manufacturer**		
GE/PHILIPS/SIEMENS/TOSHIBA	3/13/104/2	8/0/60/0	SIEMENS/ PHILIPS/MDS/Nordion/BrainLAB	113/0/7/2	12/56/0/0

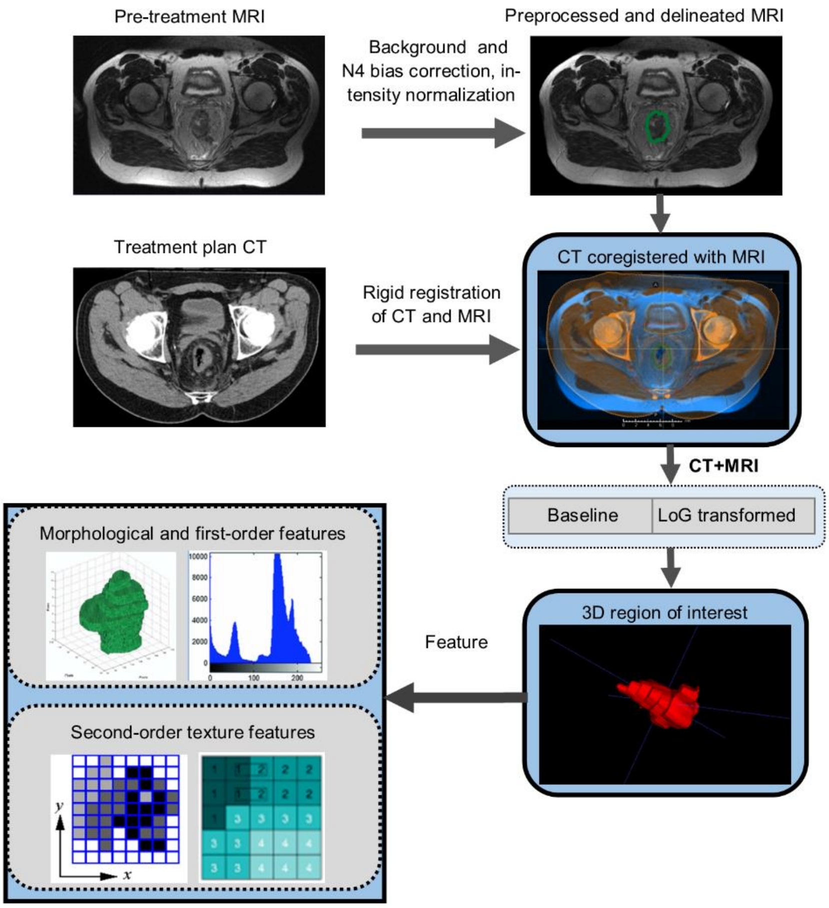

Figure S1: Image preprocessing and feature extraction pipeline. Magnetic resonance (MR) images were preprocessed and the gross tumour volume (GTV) was delineated centrally by one experienced radiation oncologist and one radiologist. GTV contours were then transferred to treatment planning computed tomography (CT) after rigid registration. All features were extracted from the GTV on the original and the Laplacian of Gaussian (LoG) transformed CT and MR images using a 3D approach.

Table S3: Image preprocessing parameters for both CT and MR data. All calculations were performed in 3D volume. Detailed configuration settings used in MIRP for MRI and CT are available at the GitHub repo. https://github.com/oncoray/radiomics-rectal_cancer

**Parameters**	**MRI**	**CT**
Pre-interpolation filter	N4 bias correction	None
Intensity normalization	95th percentile	None
Interpolated isotropic voxel spacing (mm)	1	1
Image interpolation method	linear	linear
ROI interpolation method	linear	linear
Re-segmentation range	None	[-150,180]
Merge method for texture matrices	volume merge	volume merge
Discretisation method: fixed bin number (bins)	32	32

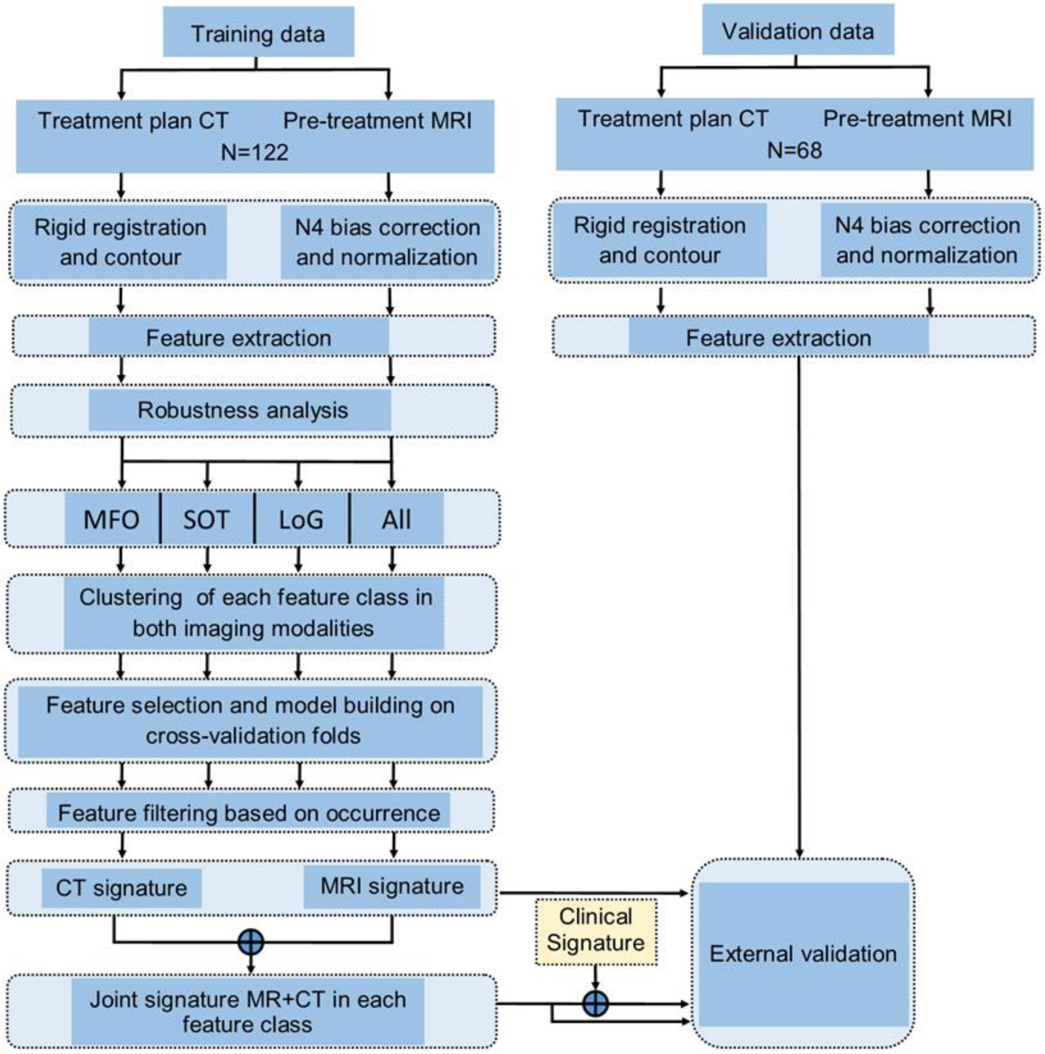

Figure S2: Modelling study workflow. After image preprocessing, radiomic features were extracted from pre-treatment T2w magnetic resonance imaging (MRI) and treatment planning computed tomography (CT) and analysed for robustness. Features were separated into morphological and first-order (MFO), second-order texture (SOT), and Laplacian of Gaussian transformed (LOG) features. Also, features in each modality were analysed without any separation to feature classes represented here by 'All'. Clustering was performed and four radiomic signatures were created individually for T2w MRI and CT based on (i) MFO, (ii) SOT, (iii) LoG, and (iv) all features using a cross-validation approach and validated independently for both endpoints. Once these signatures were developed four joint MRI and CT signatures in each feature category (i) to (iv) for both endpoints were validated with and without adding significant clinical features

Section 1: Ranking scheme for feature selection

Here we explain an example of feature selection for LoG features for tumour response prediction. The same technique applies to FFDM prediction as well. Table S4 shows fourteen Laplacian of Gaussian transformed (LoG) MRI features and fifteen CT features with the highest mutual information with tumour response selected after hierarchical clustering. These features were then used to build a prognostic model. Feature selection and model building with internal validation was first performed within 33 repetitions of 3-fold cross-validation (CV) nested in the training dataset to identify an optimal signature. Four supervised feature-selection algorithms were considered: minimal redundancy maximum relevance (MRMR) [1], mutual information maximization (MIM) [2], Elastic-net (EN) [3], and univariate logistic regression (LR). To avoid potential overfitting, only the five most relevant features were selected in each cross-validation fold. These features were then used to build a multivariable logistic-regression model on the internal training part, and validated on the internal validation part. For each of the above-mentioned feature selection methods, the occurrence of every feature in the 99 modelling steps was counted and features were ranked according to their occurrences across the cross-validation folds. Table S5 shows features with ≥50 % occurrence across each feature selection method that were further considered. Finally, features that showed repeated occurrences across at least 75% of the feature selection methods were selected (MR_log_ih_max_grad_fbn_n32 and MR_log_stat_min, CT_log_ih_max_grad_fbn_n32 and CT_log_stat_energy, Table S5). MR_log_ih_max_grad_fbn_n32 showed the highest cumulative occurrence (i.e. the highest sum of occurrences across all feature selection methods) of 365, while MR_log_stat_min showed a cumulative occurrence of 251. Both features showed a Spearman correlation of <0.5 on the entire training cohort, thus forming the MR-based LOG radiomic signature. A model with this signature was then fitted on the entire training data and the trained model was applied to the external validation data. Similarly, for CT data, CT_log_ih_max_grad_fbn_n32 showed the highest cumulative occurrence of 363, while CT_log_stat_energy showed a cumulative occurrence of 277. The features were strongly correlated with a Spearman correlation >0.5. Therefore, only CT_log_ih_max_grad_fbn_n32 was considered for the final CT-based LOG radiomic signature. The final performance in internal cross validation was considered as the average of the cross-validation training AUC (CV training) and validation AUC (CV validation). The finally selected signature and the average AUC in internal training and external validation are shown in Table S6.

Table S4: Laplacian of Gaussian transformed intensity features selected on the training cohort after clustering (left) extracted from MRI and (right) extracted from CT images. Feature definitions can be found in the IBSI reference manual [5]. MRI: magnetic resonance imaging, CT: computed tomography, LoG: Laplacian of Gaussian

MR LoG features	CT LoG features
log_loc_peak_loc	log_stat_range
log_loc_peak_glob	log_stat_median
log_stat_rms	log_stat_rmad
log_stat_var	log_ivh_i90
log_stat_skew	log_ih_kurt_fbn_n32
log_ih_rmad_fbn_n32	log_stat_p10
log_stat_min	log_stat_p90
log_stat_p90	log_stat_max
log_stat_max	log_stat_energy
log_stat_cov	log_ivh_v25
log_ih_max_grad_fbn_n32	log_ivh_v75
log_ivh_v25	log_ivh_diff_v25_v75
log_ivh_v50	log_ih_iqr_fbn_n32
log_ivh_i25	log_ih_qcod_fbn_n32
	log_ih_max_grad_fbn_n32

Table S5: Median AUC for tumour response prognosis for LoG intensity features based on MRI and CT using cross-validation of the training data. Features with an occurrence ≥50% are shown here. Features with a repeated occurrence across at least 75% (3 out of 4) of the feature selection methods are presented in bold. AUC: area under the curve, CV: cross-validation, CT: computed tomography, EN: Elastic net, LR: logistic regression, LoG: Laplacian of Gaussian, MRMR: minimum redundancy maximum relevance, MIM: mutual information maximization, MRI: magnetic resonance imaging.

Modality	Feature selection	CV training AUC	CV Validation AUC	Features	Occurrence	Cumulative occurrence of selected features
MRI	**MRMR**	0.68	0.58	**log_ih_max_grad_fbn_n32**	73	**log_ih_max_grad_fbn_n32=365**<br>**log_stat_min** =251<br><br>Remarks: Both features occurred in at least 3 out of 4 (75%) feature selection methods. Both were weakly correlated so they were considered for the MRI_LoG signature
	**MIM**	0.70	0.57	**log_ih_max_grad_fbn_n32**<br>**log_stat_min**<br>log_stat_max	98<br>92<br>60	
	**EN**	0.72	0.56	**log_ih_max_grad_fbn_n32**<br>**log_stat_min**<br>log_ivh_v25	98<br>73<br>50	
	**LR**	0.70	0.58	**log_ih_max_grad_fbn_n32**<br>**log_stat_min**<br>log_stat_max	96<br>86<br>56	
CT	**MRMR**	0.71	0.67	**log_ih_max_grad_fbn_n32**	70	**log_ih_max_grad_fbn_n32=363**<br>**log_stat_energy=277**<br><br>Remarks: Both features occurred in at least 3 out of 4 (75%) feature selection methods. Both were correlated with a Spearman correlation >0.5, therefore only **log_ih_max_grad_fbn_n32** was considered for the **CT_LoG signature**.
	**MIM**	0.73	0.62	**log_ih_max_grad_fbn_n32**<br>**log_stat_energy**<br>log_stat_median<br>log_ivh_diff_v25_v75	98<br>97<br>87<br>61	
	**EN**	0.74	0.64	**log_ih_max_grad_fbn_n32**<br>**log_stat_energy**	99<br>86	
	**LR**	0.72	0.63	**log_ih_max_grad_fbn_n32**<br>**log_stat_energy**<br>log_stat_median<br>log_ivh_diff_v25_v75	96<br>94<br>64<br>51	

Table S6: Example of average model performance computation in internal training and validation. CV: cross-validation, AUC: area under a curve, LoG: Laplacian of Gaussian

Modality	Feature level	CV training	CV validation	Signature	Final training	External validation
**MRI**	**LoG**	0.70	0.57	MR_log_ih_max_grad_fbn_n32<br>MR_log_stat_min	0.67<br>(0.57-0.75)	0.66<br>(0.51-0.82)
**CT**	**LoG**	0.73	0.64	CT_log_ih_max_grad_fbn_n32	0.70<br>(0.60-0.79)	0.61<br>(0.44-0.76)

Table S7: Univariable analysis of tumour response (logistic regression) and freedom from distant metastases (FFDM, Cox regression) in the training data. ci: confidence interval. Significant p-values are marked in bold.

**Clinical feature**		**Tumour response**		**FFDM**	
		**Odds ratio (95% ci)**	**p-value**	**Hazard ratio (95% ci)**	**p-value**
**Age** / years		1.00 (0.97-1.03)	0.92	1.00 (0.96-1.04)	0.98
**Gender** (female vs. male)		1.54 (0.69-3.38)	0.29	1.30 (0.52-3.24)	0.57
**UICC stage** (3 vs 2)		2.85 (0.46-54.78)	0.34	0.45 (0.10-1.95)	0.29
**Grade**	(1 vs0)	1.00 (0.04-14.01)	1.00	*	
	(2 vs 0)	1.92 (0.44-13.38)	0.43	1.58 (0.20-12.34)	0.66
	(3 vs 0)	2.00 (0.42-14.64)	0.42	2.45 (0.31-19.56)	0.40
**Localization**	(1 vs 0)	1.11 (0.50-2.46)	0.79	0.82 (0.32-2.12)	0.68
	(2 vs 0)	4.84 (0.4-58.06)	0.21	1.89 (0.24-14.63)	0.54
**cT**	(3 vs 2)	0.24 (0.03-1.31)	0.11	*	
	(4 vs 2)	0.06 (0.004-0.51)	**0.02**	*	
**cN** (1,2 vs 0)		*		0.47 (0.11-2.04)	0.31
**Dose** / Gy		0.87 (0.74-1.02)	0.09	0.92 (0.76-1.12)	0.42
**Chemotherapy**	(2 vs 1)	0.91 (0.22-3.82)	0.90	1.061 (0.24-4.65)	0.94
	(3 vs 1)	0.35 (0.04-3.14)	0.35	*	
	(4 vs 1)	1.28 (0.28-5.78)	0.75	0.86 (0.11-6.52)	0.89

Chemotherapy: 1 = 5 fluorouracil (FU), 2=5FU+oxaliplatine, 3= capecitabine (CAP) , 4=CAP+other
Localization (cm): 0 = 3-6, 1= > 6-12, 2= >12-16
*: The model did not converge.

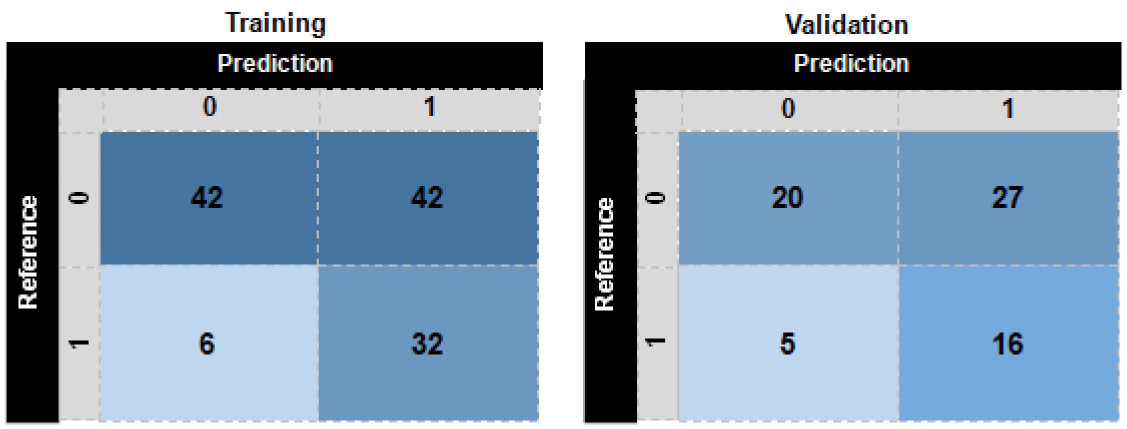

Figure S3: Confusion matrix for the prediction of tumour response to nCRT in LARC patients for the training and validation dataset at an optimal threshold of 0.42 combining clinical T stage and LoG features from MRI and CT.

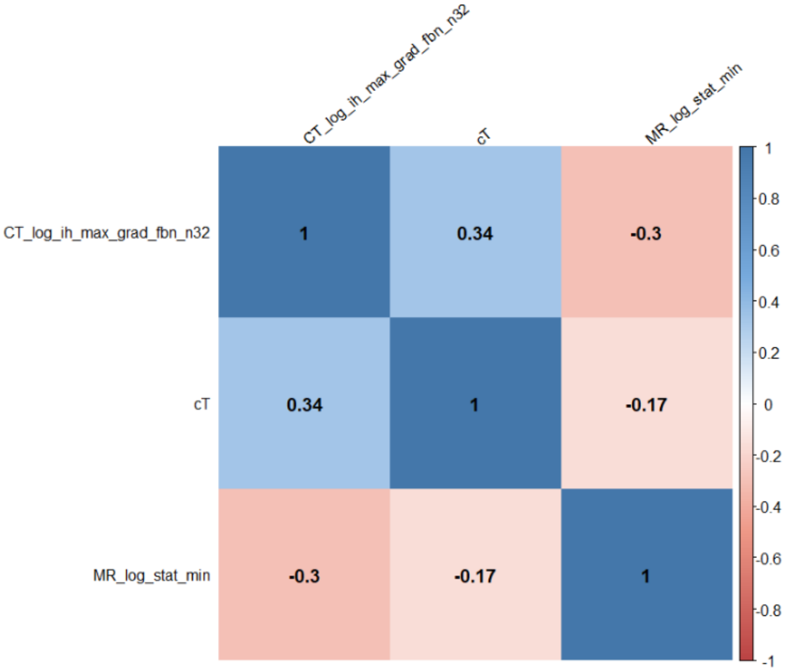

Figure S4: Correlation plot of finally selected features in the best performing clinical-radiomic signature for prediction of tumour response to nCRT. Selected features were independent predictors as shown by their low correlations, $\rho < 0.5$.

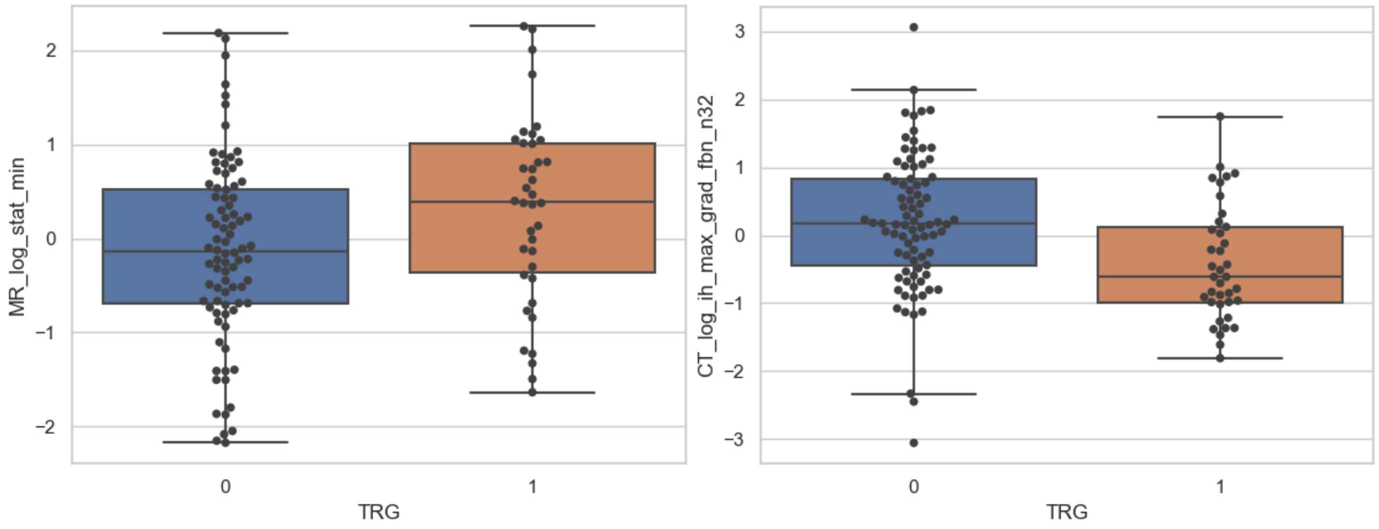

Figure S5: Box plot of Yeo-Johnson transformed and z-score normalized features selected in best performing joint CT and MRI model in training data. MRI_log_stat_min showed relatively higher values, while CT_log_ih_max_grad_fbn_n32 showed relatively lower values in responders as compared to non-responders.

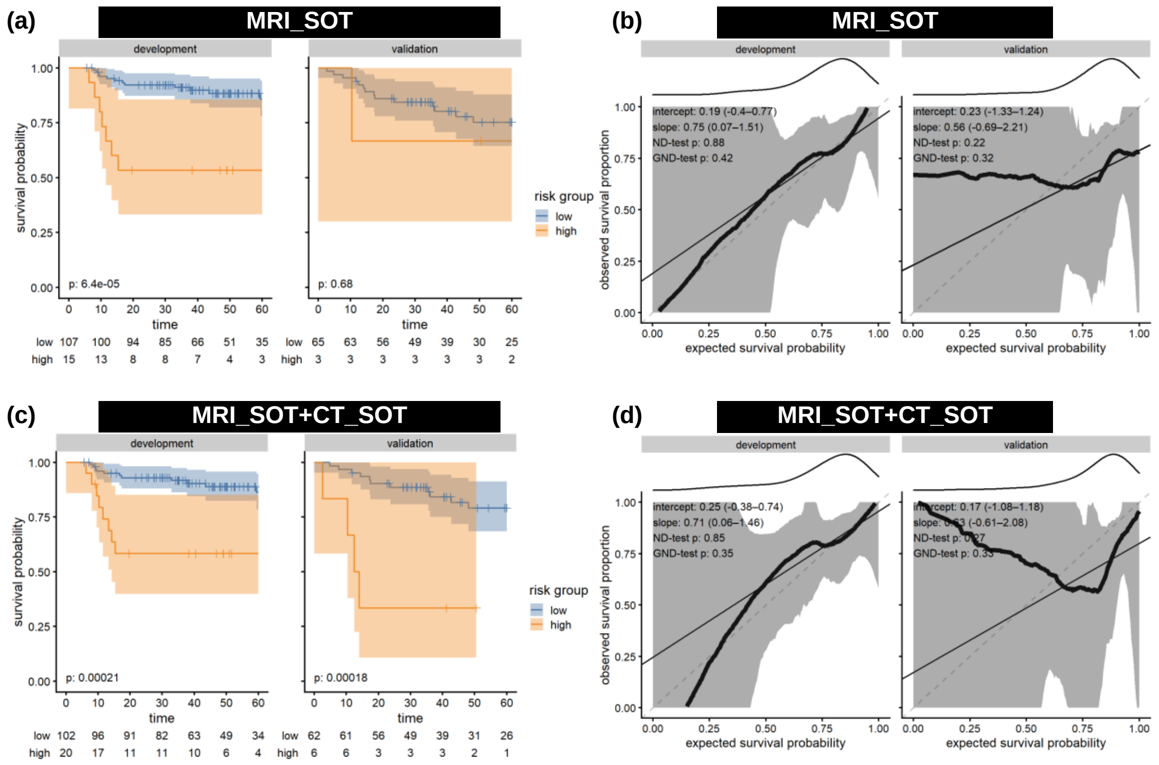

Figure S6: Kaplan-Meier curves and corresponding calibration plots for the best performing SOT signatures for the prediction of FFDM using (a,b) MRI_SOT only model, (c-d) joint MRI_SOT+CT_SOT model as presented in Table 3 of the manuscript.

Table S8: Summary of 3 selected SOT signatures from CT, MRI, and CT+MRI for the FFDM prediction. GLSZM: grey level size zone matrix, NGLDM: neighbouring grey level dependence matrix, GLCM: grey level co-occurrence matrix. 3d_fbn_n32: Features computed from discretised image intensities with fixed bin number 32 from 3D volume. d1: Chebyshev distance=1 around a central voxel for determining neighbourhood in NGLDM and GLCM based features, a0.0: alpha level 0.0 for NGLDM based features.

Signature	Features	Identifier	Texture feature type	Definition
**CT_SOT**	szm_zsnu_3d_fbn_n32	4JP3	GLSZM	This feature assesses the distribution of zone counts over the different zone sizes. Zone size non-uniformity is low when zone counts are equally distributed along zone sizes
**MRI_SOT**	ngl_dc_var_d1_a0.0_3d_fbn_n32	DNX2	NGLDM	This feature estimates the variance in dependence counts over the different possible dependence counts
	szm_sze_3d_fbn_n32	5QRC	GLSZM	This feature emphasises small zones.
	cm_clust_prom_d1_3d_v_mrg_fbn_n32	AE86	GLCM	This feature describes cluster prominence
**CT_SOT + MRI_SOT**	CT_ szm_zsnu_3d_fbn_n32	As above		
	MR_ ngl_dc_var_d1_a0.0_3d_fbn_n32			
	MR_ szm_sze_3d_fbn_n32			
	MR_ cm_clust_prom_d1_3d_v_mrg_fbn_n32			

Table S9: Final models for the prognosis of tumour response and freedom from distant metastases (FFDM). Training was performed on the entire training cohort using multivariable logistic regression for tumour response and Cox regression for

freedom from distant metastases. In addition, transformation parameters from the Yeo-Johnson transformation and z-normalization, and optimal cutoff values for Youden indexand Kaplan-Meier estimates are given. ci = confidence interval. The R models for prospective use are available on GitHub: https://github.com/oncoray/radiomics-rectal_cancer

**Tumour response**						
**Model**	**Feature**	**Coefficient**	**p-value**	**Yeo-Johnson (λ)**	**z-score normalization (mean, sigma)**	**Cutoff**
Clinical+ MRI_LoG +CT_LoG	MRI_log_stat_min	0.4282	0.027	3.3	(-0.35, 0.054)	0.248
	CT_log_ih_max_grad_fbn_n32	- 0.3088	0.004	0.0	(7.75, 1.12)	
	cT (3 vs 2)	-0.4666	0.017	-	-	
	cT (4 vs 2)	-1.2655				
	Intercept	-0.4337		-	-	
Clinical+ MRI_LoG	MRI_log_stat_min	0.3770	0.027	3.3	(-0.35, 0.054)	0.258
	MR_log_ih_max_grad_fbn_n32	-0.3221	0.008	0.1	(10.33, 2.311)	
	cT (3 vs 2)	-0.5255	0.017	-	-	
	cT (3 vs 4)	-1.3132				
	Intercept	-0.3742		-	-	
Clincial+ CT_LoG	CT_log_ih_max_grad_fbn_n32	-0.4422	0.004	0.0	(7.75, 1.12)	0.321
	cT (3 vs 2)	-0.5802	0.017	-	-	
	cT (3 vs 4)	-1.4047				
	Intercept	-0.3032		-	-	
**Freedom from distant metastases**						
CT_SOT+ MRI_SOT	MR_ ngl_dc_var_d1_a0.0_3d_fbn_n32	-0.4945	0.071	0.6	(7.85,1.81)	2.249
	MR_ szm_sze_3d_fbn_n32	-0.5044	0.192	10.0	(5.05,2.12)	
	MR_cm_clust_prom_d1_3d_v_mrg_fbn_n32	0.3013	0.176	-0.2	(4.27,0.10)	
	CT_szm_zsnu_3d_fbn_n32	-0.4584	0.046	0.3	(24.78,6.82)	
MRI_SOT	MR_ ngl_dc_var_d1_a0.0_3d_fbn_n32	-0.6337	0.071	0.6	(7.85,1.81)	2.251
	MR_ szm_sze_3d_fbn_n32	-0.4769	0.192	10.0	(5.05,2.12)	
	MR_cm_clust_prom_d1_3d_v_mrg_fbn_n32	0.2189	0.176	-0.2	(4.27,0.10)	
CT_SOT	CT_szm_zsnu_3d_fbn_n32	-0.4790	0.046	0.3	(24.78,6.82)	1.663

Section 2

Radiomics external validation study

Included studies

The prospective single centre study by De Cecco et al. [4, 5] extracted first-order intensity features from the tumour ROI delineated on the largest slice for the prediction of pathological complete responders (TRG=4) and non-responders (TRG=0-3). Images were transformed using the SSF (Laplacian of Gaussian) filter. The study reported SSF4 kurtosis to be significant in predicting response groups. To replicate this study, we extracted features from the largest tumour slices in our pooled cohort. Images were transformed using LoG filter and validation was performed for stat_kurt (IBSI: IPH6) using Wilcoxon rank-sum test and computing AUC. We adapted TRG status to match the study (TRG=4 vs TRG<4 following Dworak et al. [6]).

The study by Chidambaram et al. [7] was not strictly radiomics-based, as no high dimensional features were extracted from imaging data. However, this study analysed some basic morphological and statistical features for tumour response prediction using ADC maps of T2w MRI. Pre-treatment MRI volume was found to be significantly associated with tumour response (complete responders vs incomplete\non-responders following AJCC). We extracted morph_vol (IBSI: RNU0) from 3D GTV on the pooled cohort and analysed its significance via the Mann–Whitney-U test for tumour response (TRG=4 vs TRG<4 following Dworak et al. [6]).

In a retrospective study conducted by Caruso et al. [8] on a small cohort of 8 patients, the directional GLCM (no discretization mentioned) features extracted from T2w MRI were shown to be significantly different between pathological complete and incomplete responders, while partial responders were excluded from the study. Logistic regression model followed by Wald test for feature importance was used to analyse the predictive performance of features in the model. For validation, we excluded partial responders from our pooled training and validation data thus including only 65 patients (TRG=4 vs TRG=0 following

Dworak et al. [6]). Directional features are not supported by MIRP thus we extracted 2D GLCM features using the average method, i.e. features were computed from all matrices and then averaged using fixed bin number 64. Validation was performed by fitting a multivariable logistic regression model followed by the Wald test to obtain p-values for each feature. IBSI synonyms for validated features are mentioned in Table S10.

Two multicentre retrospective studies (Cusumano et al. [9] and Dinapoli et al. [10]) have proposed statistical and intensity histogram features extracted from LoG transformed images together with clinical T and N stage for tumour response prediction (TRG=1 vs TRG>1, Mandard et al. [11]). The signature presented by Cusumano et al. also included an additional fractal feature that could not be extracted by MIRP. The studies did not report discretization for intensity histogram features. Final model coefficients were reported in the studies. To validate the study by Cusumano et al. pixel intensities inside GTV were normalized by 99th percentile. The fractal feature was excluded, and the remaining features were extracted from 2D slices from discretized intensity histogram (25 bins). The model provided in each study was then applied on our pooled cohort. Details of features and their corresponding IBSI synonyms are presented in Table S10.

The study by Meng et al. [12] analysed statistical and intensity histogram features extracted from the largest tumour slices for tumour response prediction. The image intensities were discretized. However, the study did not report the number of bins used for discretization. Further response groups were created as responders (TRG=1-2) and non-responders (TRG=3-5) following Mandard et al. [11]. For validation, we extracted intensity histogram features from the largest tumour slices after discretizing image intensities into 25 uniform bins from pooled cohort. Finally, ih_kurt (IBSI: C3I7) feature was tested for tumour response prediction (TRG=3-4 vs TRG=0-2, Dworak et al. [6]) using the Mann-Whitney-U test.

The study by Cui et al. [13] used mpMRI to develop a radiomic signature. However, a standalone T2w MRI signature comprising directional GLCM, statistical, and morphological features extracted from the GTV for tumour response prediction (TRG=1 vs TRG>1 following Mandard et al. [11]) was also presented. The study did not report discretization, merge method, and feature extraction plane, i.e. 2D\3D for GLCM features. The study reports the coefficient of the final model built on z-score normalized features. Since directional features are not supported by MIRP, we extracted 3D GLCM features with a fixed bin number of 64 using the average method, i.e. features were computed from all matrices and then averaged, thus compensating for directional texture features. We then applied the model coefficients provided in study to compute the Radscore and AUC on our pooled cohort. Since we can get only closely related features for this study, we also fitted the logistic regression model on the train data and applied it to the validation dataset. However, this validation was not successful (AUC: train\valid = 0.72\0.32). We excluded 'HaralickCorrelation_angle90_offset7' from the signature, as by definition Haralick features are no different from GLCM 'Correlation_angle135_offset7' except for the difference in angle [14].

A retrospective study conducted by Antunes et al. [15] has reported 4 (1 Haralick co-occurrence,2 Gradient organization, 1 Laws energy response) features extracted from the largest tumour slice for tumour response prediction (TRG=4 vs TRG<4 following Dworak et al. [6]). Pre-processing applied before feature extraction in this study includes (i) image interpolation $0.781 \times 0.781 \times 4.0$ mm, (ii) N4 bias correction, and (iii) intensity normalization reference to the mean intensity of the obturator internus muscle. To validate this study, we replicated steps (i) and (ii) of pre-processing, while for step (iii) relative range intensity normalization [0, 90] was performed. Furthermore, gradient organization features are not IBSI compliant. Therefore, none of the organization features could be extracted. Finally, a random forest model was created on training data using one feature, i.e. Skewness-Laws Wave-Ripple ws = 5, and subsequently transferred to the validation data. Feature importance was computed via the Mann-Whitney-U test.

Petkovska et al. [16] has reported 6 features (2 morphological, 2 grey level texture, 2 directional Gabor) extracted from the 3D tumour volume using T2w MRI. In the study, images and corresponding tumour mask were interpolated $1 \times 1 \times 1$ mm prior to feature extraction. The study reported discretisation of normalized image intensities using fixed bin size=128, however normalization step was not clearly explained. To unambiguously specify Gabor filters at least two out of three parameters are required (scale (sigma), wavelength (lambda), bandwidth). The study reported only sigma values for Gabor filters. Model coefficient were provided in study for T2w signature. In our validation analysis we interpolated MR images to isotropic 1mm resolution using cubic interpolation followed by standard normalization of image intensities within soft tissue region. Grey level intensities were discretized using fixed bin size=128 for texture features. Gabor transformed features were extracted using angle and sigma values as reported in study however we used lambda=4 to complete feature extraction. Finally Radscore was computed by applying model coefficients using our pooled cohort and subsequently AUC was computed for tumour response prediction (TRG=4 vs TRG <4 following Dworak et al. [6]). Figure S7 shows the calibration plot for validation.

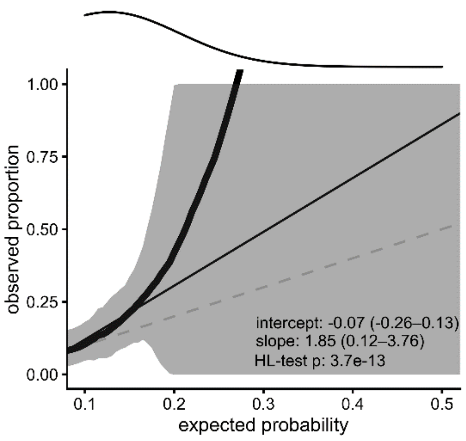

Figure S7: Calibration plots for the study by Petkvoska et al. [16]

A retrospective single-center study conducted by Petresc et al. [17] proposed a signature comprising second-order texture features on LoG and wavelet transformed images to predict tumour response (TRG=3 vs TRG=1,2 following Rayan et al.). Image intensities within the GTV were discretized using a fixed-bin width of 5. However, discretization of wavelet features, merge method for texture features, and neighbourhood distance for GLCM features was not documented. Model coefficients were provided in the study. In our validation analysis, we applied pre-processing steps as indicated in the study including image standardization (mean=0, std=100), B-spline interpolation, re-segmentation of segmentation mask. For feature extraction we used fixed bin number=64, fixed bin size=5, merge method=average, and GLCM neighbourhood distance=1. Further, we exclude the ‘wavelet_hhl_glcm_MCC’ feature as it is not standardized by IBSI. For the remaining features, we computed a Radscore by applying model coefficients on z-score normalized features using our pooled cohort and subsequently computed the AUC for tumour response prediction (TRG=3,4 vs TRG=0-2 following Dworak et al. [6]).

Table S10: Details of validated studies. ROI: Region of interest for feature extraction, NA: not applicable, NS: Not supported

Study	Grading scheme	ROI	Image processing in study	Image processing applied for validation	Feature	IBSI synonyms	Remarks
De Cecco (2015)	AJCC	Largest	SSF filtering	LoG filter at sigma =4mm	(i) Kurtosis	(i) stat_kurt	The feature extraction pipeline can be completely implemented.
De Cecco (2016)	AJCC	Largest	SSF filtering	LoG filter at sigma=4mm	(i) Kurtosis<br>(ii) Ve from pMRI	(i) stat_kurt<br>(ii) NS	We validated only the first feature.
Chidbaram (2017)	AJCC	3D	NA	NA	(i) Tumour volume	(i) morph_volume	Replicated completely.
Caruso (2018)	Grading system was not mentioned in study	2D	NA	NA	GLCM at 0˚, 45˚, 90˚, 135˚<br>(i) Energy<br>(ii) Contrast<br>(iii) Correlation<br>(iv) Inverse difference momentum or homogeneity<br>(v) Entropy	(i) cm_energy<br>(ii) cm_contrast<br>(iii) cm_corr<br>(iv) cm_inv_diff<br>(v) cm_joint_entr	Features extracted using average merge method for all texture metrics, thus including information from all directions.
Meng (2018)	Mandrad	Largest	Voxel intensities were discretized	Voxel intensities were discretized By fixed bin number =25	(i) kurtosis	(i) ih_kurtosis	The study did not mention number of bins used for discretization. We discretized image intensities to 25 bins.
Cusumano (2018)	Mandard	2D	(i) Intensity normalization by 99th percentile within GTV<br>(ii) LoG filter	(i) Intensity normalization by 99th percentile within GTV<br>(ii) LoG filter	(i) cT<br>(ii) cN<br>(iii) Entropy (LoG σ=0.34)<br>(iv) Skewness (LoG σ=0.48)<br>(v) Max Fractal Dimension (FD) (40-100)	(iii) ih_entropy (LoG σ=0.34)<br>(iv) stat_skew (LoG σ=0.48)<br>(v) NS	Feature (v) cannot be extracted, clinical T and N stage were also included in validation analysis, model parameter used from study for feature (i)–(iv).
Dinapoli (2018)	Mandard	2D	(i) LoG filter	(i) LoG filter	(i) cT<br>(ii) cN<br>(iii) Entropy (LoG σ=0.344)<br>(iv) skewness (LoG σ=0.485)	(iii) ih_entropy (LoG σ=0.344)<br>(iv) stat_skew (LoG σ=0.485)	Clinical T and N stage were also included in validation analysis, model parameter used from study.
Cui (2018)	Mandard	NA	(i) Features normalization	(i) Features normalization	(i) kurtosis<br>(ii) ClusterProminence _AllDirection_offset7_SD<br>(iii) InverseDifference Moment_angle0_offset7<br>(iv) GLCMEnergy_angle45 _offset7<br>(v) HaralickCorrelation_ angle90_offset7<br>(vi) Correlation_angle135 _offset7<br>(vii) ClusterShade_angle	(i) stat_kurt<br>(ii) cm_clust_prom<br>(iii) cm_inv_diff_mom<br>(iv) cm_energy<br>(v) NA<br>(vi) cm_corr<br>(vii) cm_clust_shade<br>(viii) morph_sph_dispr	We validated T2w signature by extracting non-directional features using 3D ROI using fixed bin number discretization = 32 bins and merge method = average

					135_offset7<br>(viii) SphericalDisproportion		We excluded 'HaralickCorrelation' from the signature, as by definition Haralick features are no different from non-directional GLCM 'Correlation' features.<br>Model parameters used form study for validation.
Antunes (2020)	Dworak	Largest	(i) Interpolation= 0.781 × 0.781 × 4.0 mm<br>(ii) N4 bias correction<br>(iii) Intensity normalization reference to the mean intensity of the obturator internus muscle	(i) Interpolation= 0.781 × 0.781 × 4.0 mm<br>(ii) N4 bias correction<br>(iii) Intensity normalization within the range of 0.0-0.90	(i) Skewness-Laws Wave-Ripple ws = 5<br>(ii)Kurtsosis-Haralick SumEntropy ws = 9<br>(iii) Skewness-CoLlAGe Correlation ws = 5<br>(iv)Kurtosis-CoLlAGe InformationMetric1 ws = 3	(i) stat_skew (on energy map of the W5R5 Laws kernel)<br>(ii) NS<br>(iii) NS<br>(iv) NS	Our data does not contain delineation for obturator internus muscle. Therefore, in order to replicate image processing step (iii), relative range intensity normalization was performed within masked image.<br>Organization feature are not IBSI compliant, therefore none of organization features could be validated.
Petkvoska (2020)	Histopathology	3D	(i) Interpolation= 1 × 1×1 mm<br>(ii) Normalized voxel intensities were discretized (normalization was not explained)<br>(ii) Gabor filter	(i) Interpolation=1 × 1×1 mm<br>(ii) Standard normalization<br>(iii) Voxel intensities were discretized<br>(ii) Gabor filter	(i) shape surface area<br>(ii) shape compactness<br>(iii) GLCM difference variance<br>(iv) GLSZM size zone low-gray level emphasis<br>(v) std of Gabor (sigma=2 theta=30)<br>(vi) kurtosis of Gabor (sigma=2sqrt2, theta=30)	(i) morph_area<br>(ii) morph_comp_1<br>(iii) cm_diff_var<br>(iv) szm_lgze<br>(v) $\sqrt{\text{stat. var}}$ (Gabor, σ =2, $\lambda = 4,$ theta=30)<br>(vi) stat.kurt (Gabor ,sigma=2sqrt2, $\lambda = 4,$ theta=30)	Normalization process was not clearly mentioned in study therefore we used standard normalization of MRI intensities before feature extraction<br>The study did not report lambda and/or bandwidth for Gabor features. Thus, to complete feature extraction we used lambda=4.<br>Model parameters used form study for validation.

Petresc (2020)	Ryan	3D	(i) Image normalization (mean=0, std=100)<br>(ii) B-spline interpolation (x=y=z=2mm)<br>(iii) Resegmentation of segmentation mask<br>(iii) z-score normalization of extracted features before feature selection	(i) Image normalization (mean=0, std=100)<br>(ii) B-spline interpolation (x=y=z=2mm)<br>(iii) Resegmentation of segmentation mask<br>(iii) z-score normalization of extracted features before feature selection	(i) log_sigam_5.0_mm_3D_glszm_SmallAreaEmphasis<br>(ii) wavelet_lhl_glcm_correlation<br>(iii) wavelet_lhl_firstorder_10Perecntile<br>(vi) wavelet_hhl_glcm_Imc1<br>(v) wavelet_hhl_firstorder_kurtosis<br>(vi) wavelet_hhl_glszm_SmallAreaHighGrayLevelEmphasis<br>(vii) wavelet_hhl_glcm_MCC	(i) szm_sze (LoG, σ =5.0)<br>(ii) cm_corr (wavelet filter=lhl)<br>(iii) stat_p10 (wavelet filter=lhl)<br>(vi) cm_info_corr1(wavelet filter=hhl)<br>(v) stat_kurt (wavelet filter=hhl)<br>(vi) szm_szhge (wavelet filter=hhl)<br>(vii) NS	Feature (vii) is not IBSI standardized therefore model was validated using features (i)-(vi). Model parameters used form study for validation.

Table S11: Clinical characteristics of included studies, NP: information not provided in manuscript

Study	Patient number	Field strength	Sequence	Dose (Gy)	In-plane voxel dimension/ slice thickness (mm)	Male/female average or median age	Responders/ non-responders	cT/cN
De Cecco (2015)	15	3T	T2w FSE	50.4-54	NP/4.0	9/6<br>Average age = 63.3	6/9	locally advanced tumor stages II (cT3-4, N0, M0) and III (cT1-4, N+, M0)
De Cecco (2016)	12	3T	T2w FSE	50.4-54	NP/4.0	4/8<br>NP	6/6	locally advanced tumor stages II (cT3-4, N0, M0) and III (cT1-4, N+, M0)
Chidbaram (2017)	78	1.5T, 3T	T2w FSE	45-50	0.7/4.0	NP	8/51<br>For 8 patients, response was not available	cT 2/3/4/Any= 20/44/6/8<br>cN 0/1/2/Any = 18/28/24/8
Caruso (2018)	8	3T	T2w FSE	NP	NP/4.0	6/2<br>Median age 65.5	NP	cT 2/3/4 = 16/120/62<br>cN 0/1/2 = 13/67/118
Casumano (2018)	Total = 198<br>Train = 173<br>Valid = 25	NP	T2w FSE	50-55	NP	NP<br>Average age train = 63	Train 47/126	Train cT 2/3/4= 15/100/58<br>Train cN 0/1/2=10/60/103
DiNPpoli (2018)	Total = 226<br>Train = 162<br>Valid_1 = 39<br>Valid_2 = 25	1.5T	T2w FSE	45-55	0.76/NP	NP<br>Median age train = 65	Train 46/116	Train cT 2/3/4= 15/95/52<br>Train cN 0/1/2= 9/58/95
Meng (2018)	59	3T	T2w SE	50	NP/3.0	39/20<br>Average age = 54	30/29	cT 3/4 = 37<br>cN 0/+ = 22
Cui (2019)	Total = 186<br>Train = 131<br>Valid = 55	3T	NP	50	NP	Train 83/48<br>Average age train = 53	Train 22/109	Train cT 3/4 = 94/37<br>Train cN 0/1/2 = 13/71/47
Antunes (2020)	Total = 152<br>Train = 60<br>Valid = 44	1.5T,3T	T2w TSE	45-50.4	0.313-1.172/3.0-6.0	Train 50/10<br>Average age train = 63	Train 13/47	Train cT 1-2/3-4/Any= 10/46/4<br>Train cN 0/+/Any = 13/43/4
Petkvoska (2020)	102	1.5T,3T	T2w FSE	50.4	NP/2.0-4.0	60/42<br>Median age = 61	19/83	cT 2/3/4 = 9/85/8<br>vascular invasion Yes/No/Any = 20/81/1
Petresc (2020)	Total = 67<br>Train = 44<br>Valid = 23	1.5T	T2w TSE	NP	NP/3.0	Train 33/11<br>Average age train = 57.4	Train 27/17	cT 2/3/4 = 6/32/6<br>cN 1/2 = 11/33

References

1. Peng, H., F. Long, and C. Ding, *Feature selection based on mutual information criteria of max-dependency, max-relevance, and min-redundancy.* IEEE Transactions on pattern analysis and machine intelligence, 2005. **27**(8): p. 1226-1238.
2. Gel'fand, I.M. and A.M. Yaglom, *Computation of the amount of information about a stochastic function contained in another such function.* Uspekhi Matematicheskikh Nauk, 1957. **12**(1): p. 3-52.
3. Zou, H. and T. Hastie, *Regularization and variable selection via the elastic net.* Journal of the royal statistical society: series B (statistical methodology), 2005. **67**(2): p. 301-320.
4. De Cecco, C.N., et al., *Texture analysis as imaging biomarker of tumoral response to neoadjuvant chemoradiotherapy in rectal cancer patients studied with 3-T magnetic resonance.* Investigative radiology, 2015. **50**(4): p. 239-245.
5. De Cecco, C.N., et al., *Performance of diffusion-weighted imaging, perfusion imaging, and texture analysis in predicting tumoral response to neoadjuvant chemoradiotherapy in rectal cancer patients studied with 3T MR: initial experience.* Abdominal Radiology, 2016. **41**(9): p. 1728-1735.
6. Dworak, O., L. Keilholz, and A. Hoffmann, *Pathological features of rectal cancer after preoperative radiochemotherapy.* International journal of colorectal disease, 1997. **12**(1): p. 19-23.
7. Chidambaram, V., et al., *Investigation of volumetric apparent diffusion coefficient histogram analysis for assessing complete response and clinical outcomes following pre-operative chemoradiation treatment for rectal carcinoma.* Abdominal Radiology, 2017. **42**(5): p. 1310-1318.
8. Caruso, D., et al., *Haralick's texture features for the prediction of response to therapy in colorectal cancer: a preliminary study.* La radiologia medica, 2018. **123**(3): p. 161-167.
9. Cusumano, D., et al., *Fractal-based radiomic approach to predict complete pathological response after chemo-radiotherapy in rectal cancer.* La radiologia medica, 2018. **123**(4): p. 286-295.
10. Dinapoli, N., et al., *Magnetic resonance, vendor-independent, intensity histogram analysis predicting pathologic complete response after radiochemotherapy of rectal cancer.* International Journal of Radiation Oncology* Biology* Physics, 2018. **102**(4): p. 765-774.
11. Mandard, A.M., et al., *Pathologic assessment of tumor regression after preoperative chemoradiotherapy of esophageal carcinoma. Clinicopathologic correlations.* Cancer, 1994. **73**(11): p. 2680-2686.
12. Meng, Y., et al., *Novel radiomic signature as a prognostic biomarker for locally advanced rectal cancer.* Journal of Magnetic Resonance Imaging, 2018. **48**(3): p. 605-614.
13. Cui, Y., et al., *Radiomics analysis of multiparametric MRI for prediction of pathological complete response to neoadjuvant chemoradiotherapy in locally advanced rectal cancer.* European radiology, 2019. **29**(3): p. 1211-1220.
14. Zwanenburg, A., et al., *The image biomarker standardization initiative: standardized quantitative radiomics for high-throughput image-based phenotyping.* Radiology, 2020. **295**(2): p. 328-338.
15. Antunes, J.T., et al., *Radiomic features of primary rectal cancers on baseline T2-weighted MRI are associated with pathologic complete response to neoadjuvant chemoradiation: a multisite study.* Journal of Magnetic Resonance Imaging, 2020. **52**(5): p. 1531-1541.
16. Petkovska, I., et al., *Clinical utility of radiomics at baseline rectal MRI to predict complete response of rectal cancer after chemoradiation therapy.* Abdominal Radiology, 2020. **45**(11): p. 3608-3617.
17. Petresc, B., et al., *Pre-treatment T2-WI based radiomics features for prediction of locally advanced rectal cancer non-response to neoadjuvant chemoradiotherapy: a preliminary study.* Cancers, 2020. **12**(7): p. 1894.
